# Sr, Fe Co-doped Perovskite Oxides With High Performance for Oxygen Evolution Reaction

**DOI:** 10.3389/fchem.2019.00224

**Published:** 2019-04-24

**Authors:** Qiang Guo, Xiang Li, Haifei Wei, Yi Liu, Lanlan Li, Xiaojing Yang, Xinghua Zhang, Hui Liu, Zunming Lu

**Affiliations:** ^1^School of Materials Science and Engineering, Hebei University of Technology, Tianjin, China; ^2^School of Materials Science and Engineering, Tianjin University, Tianjin, China

**Keywords:** La_0.4_Sr_0.6_Ni_0.5_Fe_0.5_O_3_, perovskite, oxygen evolution reaction, charge transfer resistance, *e*_*g*_ filling

## Abstract

Developing efficient and earth-abundant electrocatalysts for the oxygen evolution reaction (OER) is still a big challenge. Here, perovskite La_0.4_Sr_0.6_Ni_0.5_Fe_0.5_O_3_ nanoparticles were rationally designed and synthesized by the sol-gel method with an average size around 25 nm, and it has a remarkable intrinsically activity and stability in 1 M KOH solution. Compared with other perovskite (LaNiO_3_, LaFeO_3_, and LaNi_0.5_Fe_0.5_O_3_) catalysts, La_0.4_Sr_0.6_Ni_0.5_Fe_0.5_O_3_ exhibits superior OER performance, smaller tafel slope and lower overpotential. The high electrochemical performance of La_0.4_Sr_0.6_Ni_0.5_Fe_0.5_O_3_ is attributed to its optimized *e*_*g*_ filling (~1.2), as well as the excellent conductivity. This study demonstrates co-doping process is an effective way for increasing the intrinsic catalytic activity of the perovskite.

## Introduction

Oxygen electrocatalyst plays a very important role in oxy-renewable energy technologies (Zhu et al., 2015a), such as rechargeable metal-air batteries, regenerative fuel cells and water splitting. Recently, the development of highly active oxygen evolution reaction (OER) catalysts in alkaline solutions has become a hot topic of electrocatalytic water splitting technology (Gupta et al., 2016; Zhao et al., 2017). However, the OER at the anode of the water electrolyze is hindered by the kinetics of the complex four-electron oxidation process, which requires a considerable overpotential (η), leading to a significant decrease in the overall efficiency of water splitting (Jiao et al., 2015). In order to acquire high reaction kinetic and low overpotential in practical applications, noble metal oxides are usually used as catalysts (such as IrO_2_ and RuO_2_), but the high cost and scarcity of noble metals restrict their large-scale commercialization.

More recently, non-noble metal perovskite oxide (ABO_3_) have been extensively investigated as OER catalysts for their electronic adjustability and flexibility in physical and chemical properties (Jin et al., 2011; Grimaud et al., 2013; Hong et al., 2015; Hwang et al., 2017). For instance, Suntivich et al. reported the rational design of a descriptor with a high OER perovskite electrocatalyst, that is the intrinsic activity of ORR (oxygen reduction reaction)/OER (Jin et al., 2011; Suntivich et al., 2011) in alkaline solutions can be enhanced when the high energy anti-bonding orbital *e*_*g*_ of the B-site transition metal in the perovskite oxides is occupied close to unity. This is because the number of the electrons in the *e*_*g*_ orbits of B-site transition metal can greatly influence the bonding of oxygen-containing intermediate, especially for OH^*^, during OER process, and thus optimizing the OER performance (Suntivich et al., 2011). Based on this theory, they obtained a highly efficient dual-function perovskite electrocatalyst Ba_0.5_Sr_0.5_Co_0.8_Fe_0.2_O_3−δ_. Its performance surpasses that of the most active IrO_2_ catalyst in alkaline media (Suntivich et al., 2011). After these two pioneering woks, many high efficient perovskite catalysts were obtained when the *e*_*g*_ filling of the B-site transition metal was adjusted to 1.2, which is served as the optimal value for high performance (Petrie et al., 2016; Zhou et al., 2016; Retuerto et al., 2017; Tong et al., 2017), through the regulating the grain size (Zhou et al., 2016; Retuerto et al., 2017), the lattice mismatch at the interface (Petrie et al., 2016; Tong et al., 2017) and co-doping of cations (Tiwari et al., 1996; Ge et al., 2016; Raabe et al., 2016; Chen et al., 2017). Among these methods, co-doping of cations is the most efficient way for adjusting the *e*_*g*_ filling to enhance the electrochemical performance of perovskite. Especially based on the Shao-Horn's researches, the doping of B-site metal can effectively adjust the *e*_*g*_ filling of perovskite oxides. Recently, Zhu et al. (2015b) achieved high OER activity by using Nb partial substitution of the B-site Co ions in SrCo_0.8_Fe_0.2_O_3_ to adjust the *e*_*g*_ filling to ~1.2. However, only B-site metal doping usually deviate from the optimal *e*_*g*_ filling (Guo et al., 2015; Tong et al., 2017). The partial substitution of A-site metal ion with a valence state of +2 or +1 is an effective way for amending the deviation (Mefford et al., 2016). Furthermore, the doping of A-site can also enhance the electrical conductivity of the catalyst (Mefford et al., 2016; Yan et al., 2017).

Herein, we designed a series of La_1−y_Sr_y_Ni_1_-_x_Fe_x_O_3_ (x = 0, 0.1, 0.3, 0.5, 0.7, 1; y = 0.2, 0.4, 0.6) by co-doping LaNiO_3_ parent oxide with Fe and Sr to optimizing *e*_*g*_ filling and enhance conductivity. By tuning the ratio of La/Sr and Ni/Fe, we can maximize the LaNiO_3_ OER performance. The particle size of La_0.4_Sr_0.6_Ni_0.5_Fe_0.5_O_3_ obtained in this paper is about 25 nm, which is more favorable for the OER reaction in alkaline solution (Zhou et al., 2016; Retuerto et al., 2017). The La_0.4_Sr_0.6_Ni_0.5_Fe_0.5_O_3_ exhibits an overpotential of 320 mV at 10mA cm^−2^, a tafel slope of 52.77 mV dec^−1^ and a high stability, i.e., the current density of La_0.4_Sr_0.6_Ni_0.5_Fe_0.5_O_3_ decreased only 9.3% after 10 h of continuous polarization, which is comparable with commercial RuO_2_. These electrochemical results proved that La_0.4_Sr_0.6_Ni_0.5_Fe_0.5_O_3_ is a promising OER reaction electrocatalyst that can be applied in fuel cell, rechargeable metal-air batteries and so on.

## Experimental Section

### Material

All chemicals (analytical reagent grade) used in this work, including La(NO_3_)_3_·6H_2_O, Sr(NO_3_)_2_·4H_2_O, Ni(NO_3_)_2_·6H_2_O, Fe(NO_3_)_2_·9H_2_O, ethylene glycol(HOCH_2_CH_2_OH, ≥99.8%), ethanol(CH_3_CH_2_OH), KOH, Citric acid[HOC(COOH)(CH_2_COOH)_2_, ≥99.5%], RuO_2_ and Nafion (15 wt%) were purchased from Sigma-Aldrich and used without further purification. Deionized water was used in all experiments.

### Material Synthesis

A series of LaNi_1−x_Fe_x_O_3_ (x = 0, 0.1, 0.3, 0.5, 0.7, 1), La_1−y_Sr_y_Ni_0.5_Fe_0.5_O_3_ (y = 0.2, 0.4, 0.6), and La_1−z_Sr_z_NiO_3_ (z = 0.2, 0.4, 0.6, 0.8) perovskite nanoparticles were prepared by sol-gel method and solid-state method reported previously (Yuasa et al., 2012; Xu et al., 2016a). Briefly, La(NO_3_)_3_·6H_2_O, Ni(NO_3_)_2_·6H_2_O, Fe(NO_3_)_3_·9H_2_O and Sr(NO_3_)_2_·4H_2_O, were dissolved in deionized water to form a 5 mL solution. Subsequently, the above metal nitrate solution was gradually added to citric acid (HOC(COOH)(CH_2_COOH)_2_, ≥99.5%) and ethylene glycol (HOCH_2_CH_2_OH, ≥99.8%) under vigorous stirring conditions at room temperature. The mixture was then heated at 80°C with water bath to form a viscous gel, and then it was heated at 200°C in oven until a xerogel formed. The obtained xerogel was sintered in a muffle furnace at 750°C for 10 h to form a perovskite oxide powder with well-crystallized structure. All the powders after heat treatment were finely ground using mortar and pestle prior to further characterization.

### Material Characterization

All the obtain perovskite oxides were characterized by X-ray diffraction (XRD, Siemens-Bruker D5000) with Cu Kα radiation. Transmission electron microscope (TEM) images, energy dispersive spectrometer, EDS mapping, were carried out on FEI Tecnai G2 F20 transmission electron microscope operated at 200 kV. X-ray photoelectron spectroscopy (XPS) analyses were performed using a PHI Quantum 2000 scanning ESCA Microprobe spectrometer, and scanning electron microscope (SEM) images were performed by a field emission scanning electron microscope model S-4800. Brunauer-Emmett-Teller (BET) surface area and pore size distribution was measured by N_2_ adsorption-desorption analysis using Quantachrome Autosorb-IQ.

### Electrode Calibration

The relation: E (RHE) = E (SCE) + 1.041 V is come from E (RHE) = E (SCE) + 0.0591^*^pH+ 0.244 V. Before the electrochemical test, we have calibrated the reference electrode of the saturated calomel electrode.

The calibration of saturated calomel electrode:

- Calibration is performed in a three-electrode system in which a platinum electrode as a counter electrode, and a saturated calomel electrode as a reference electrode. And the calibration is also performed in a H_2_ saturated 1 M KOH electrolyte.
- Cyclic voltammetry scanning was repeated at a sweep rate of 1 mV s^−1^ until the cycle curves were completely coincident.
- Draw the CV curve of the last lap as shown in Figure S5. When the current value is 0, there are two corresponding potentials. The average value of the two potentials is taken as the thermodynamic potential. Therefore, in a 1 M KOH electrolyte, the calibration formula is E (RHE) = E (SCE) + 1.041 V.

### Electrochemical Measurement

Electrochemical measurements were performed in a three-electrode electrochemical cell configuration with O_2_-saturated 1.0 M KOH at pH = 14 controlled by a CHI 750E electrochemistry workstation (Stevens et al., 2017). During the electrochemical test, a glass carbon electrode(GC) loaded with our perovskite catalysts was used as a working electrode, an Hg/Hg_2_Cl_2_(SCE) electrode in saturated KCl solution was used as a reference electrode, and a piece of Pt plate was used as a counter electrode. Before testing, we would pre-polish the GC electrode with 50 nm α-Al_2_O_3_ slurries on a polishing cloth and rinsed with deionized water. For the fabrication of catalyst inks, 5 mg perovskite oxide power and 10 mg carbon black (XC-72, which was treated by nitric acid for 5 h) were dispersed in 1 mL of mixture water and ethanol, followed by adding 40 μL nafion solution (5 wt%) for 2 h ultrasonication to form a homogeneous ink. The OER catalytic activity of nitric acid-treated carbon black (XC-72) is very weak and can be ignored (Figure S1). Then, 4.2 μL of the catalyst ink was transferred onto the surface of GC electrode, which was used as a working electrode with a yielding of 0.25 mg cm^−2^ catalyst. The OER test was performed at room temperature using a standard three-electrode in an electrolytic cell with a CHI750E bipotentiostat. During the electrochemical test, a flow of ultra-pure O_2_ was maintained over the system to ensure the O_2_/H_2_O equilibrium at 1.23 V vs. reversible hydrogen electrode (RHE). All electrochemical measurements were performed in 1M KOH solution. Prior to OER recording, the potential of the perovskite catalyst was scanned at 50 mV s^−1^ between 0 and 0.5 V vs. SCE until a stable cyclic voltammogram (CV) was recorded. The line scan voltammogram (LSV) polarization curves were tested between 0 to 0.8 V vs. SCE with a scan rate 5 mV s^−1^. Tafel slope was obtained by plotting log(J) from LSV curves. The electrochemical impedance spectroscopy was measured at 0.55 V vs. SCE with a frequency from 0.1 to 10^6^ Hz. The electrochemical surface area (ECSA) performance was derived by performing CV measurements at different scan rates of 5, 10, 15, 20, and 25 mV s^−1^ by selecting a potential range (0.1 V to 0.15 V) where the perovskite catalysts do not react. By plotting J = (J_a_-J_c_) (J_a_ and J_c_ are anode and cathode current density, respectively) at 1.125 V against the scan rates, ECSA was calculated from the linear slope that was twice of the double layer capacitance (*Cdl*). To test the stability of the perovskite catalysts, a galvanostatic measurement at a fixed current density (J) of 10 mA cm^−2^ was performed on Carbon Fiber Paper.

### Faraday Efficiency

To calculate the FE for OER, RRDE voltammogram was conducted on a RRDE configuration (Pine Research Instrumentation, USA) in N_2_-saturated 1 M KOH solution with the working electrode continuously rotating at 1600 rpm to remove oxygen bubbles. The FE was evaluated according to Equation (1) (Suntivich et al., 2011):

(1) $$\begin{array}{r} FE=\frac{I_{r}}{N\times I_{d}}\times 100\% \end{array}$$

where, I_r_ is the ring current obtained at a constant potential of 0.4 V, I_d_ the given current on the disk, N the current collection efficiency (≈ 0.2) of the RRDE.

## Results and Discussion

**Figure 1:**
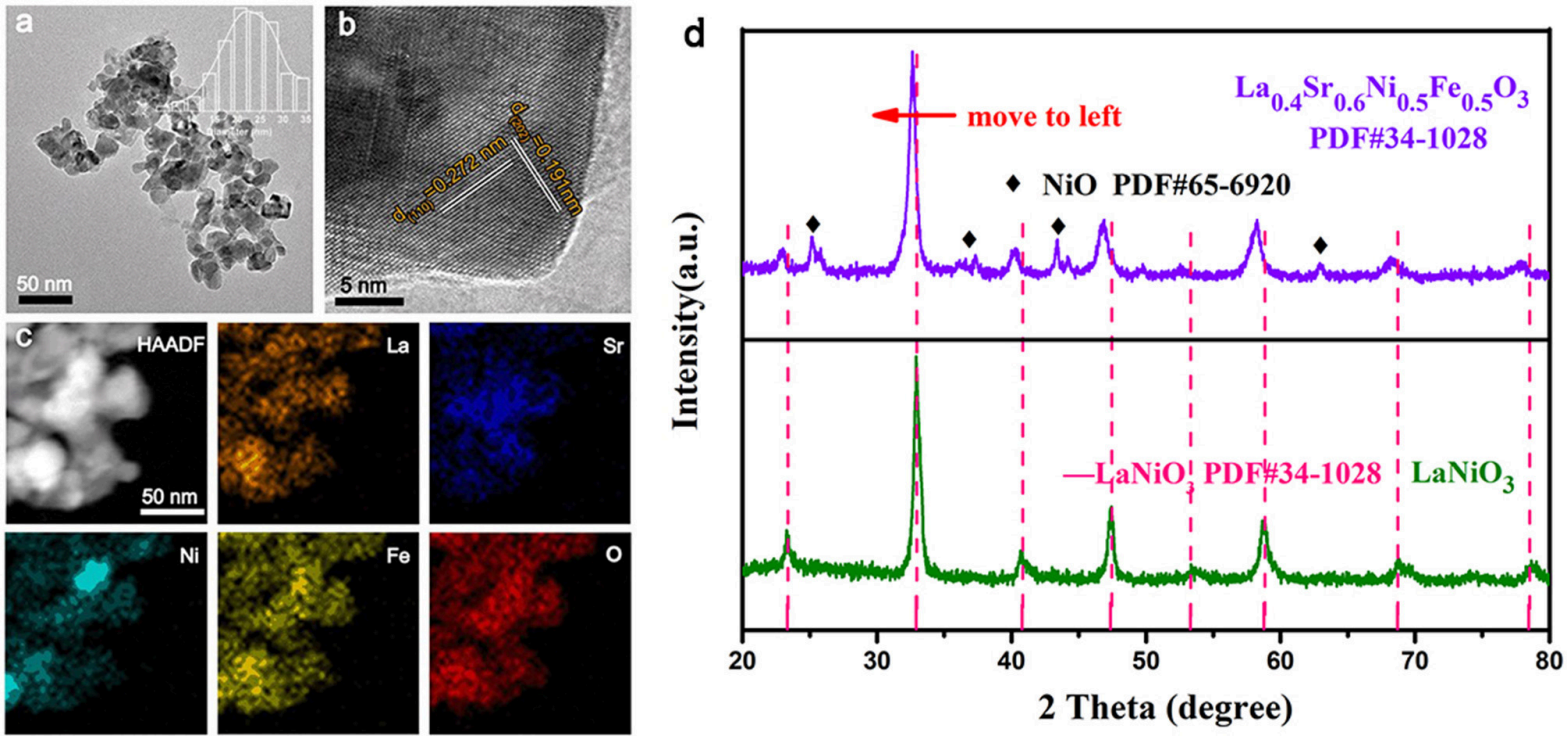
**(a)** TEM image, the inset is the particle size chart of La_0.4_Sr_0.6_Ni_0.5_Fe_0.5_O_3_ in **(a)**; **(b)** High magnification TEM image of the La_0.4_Sr_0.6_Ni_05_Fe_0.5_O_3_ nanoparticles; **(c)** HAADF image and EDS mapping of La_0.4_Sr_0.6_Ni_05_Fe_0.5_O_3_ with the elemental mapping of La, Sr, Ni, Fe, and O; **(d)** XRD patterns of LaNiO_3_ and La_0.4_Sr_0.6_Ni_05_Fe_0.5_O_3_ perovskite oxides.

As shown in the inset of Figure 1a, the crystal structure of La_0.4_Sr_0.6_Ni_0.5_Fe_0.5_O_3_ has a cubic structure with an average size of 25 nm. The high resolution TEM (HRTEM) image (Figure 1b) of a nanoparticle exhibits lattice fringes with spacings of 0.272 nm and 0.191 nm, corresponding to the (110) and (202) crystal planes of LaNiO_3_ (PDF#34-1028), respectively, indicating the structure retained after Sr and Fe co-doping. High angle angular dark field (HAADF) image (Figure 1c) and energy dispersive spectrometer (EDS) mapping (Figure S2a) reveals that the La_0.4_Sr_0.6_Ni_0.5_Fe_0.5_O_3_ nanoparticles are composed of La, Sr, Ni, Fe, and O elements, which are distributed evenly throughout the particle, suggesting that Sr and Fe successfully doping into LaNiO_3_ crystal structure (Klaus et al., 2015). The X-ray diffraction (XRD) pattern shown in Figure 1d further prove the cubic structure of the La_0.4_Sr_0.6_Ni_0.5_Fe_0.5_O_3_. Moreover, a small amount of NiO occurs during the synthesis of perovskite, and the presence of NiO does not affect the catalytic performance (Retuerto et al., 2017). Compared with LaNi_1−x_Fe_x_O_3_ and La_1−y_Sr_y_Ni_0.5_Fe_0.5_O_3_ (Figures S2b,c), the La_1−z_Sr_z_NiO_3_ (Figure S2d) cannot maintain the cubic structure. Thus, we did not discuss the electrochemical performance of La_1−z_Sr_z_NiO_3_ in subsequent electrochemical performance tests. The scanning electron microscope (SEM) showed that the morphology of LaNiO_3_ was unchanged after Fe and Sr co-doping (Figures S3, S4).

**Figure 2:**
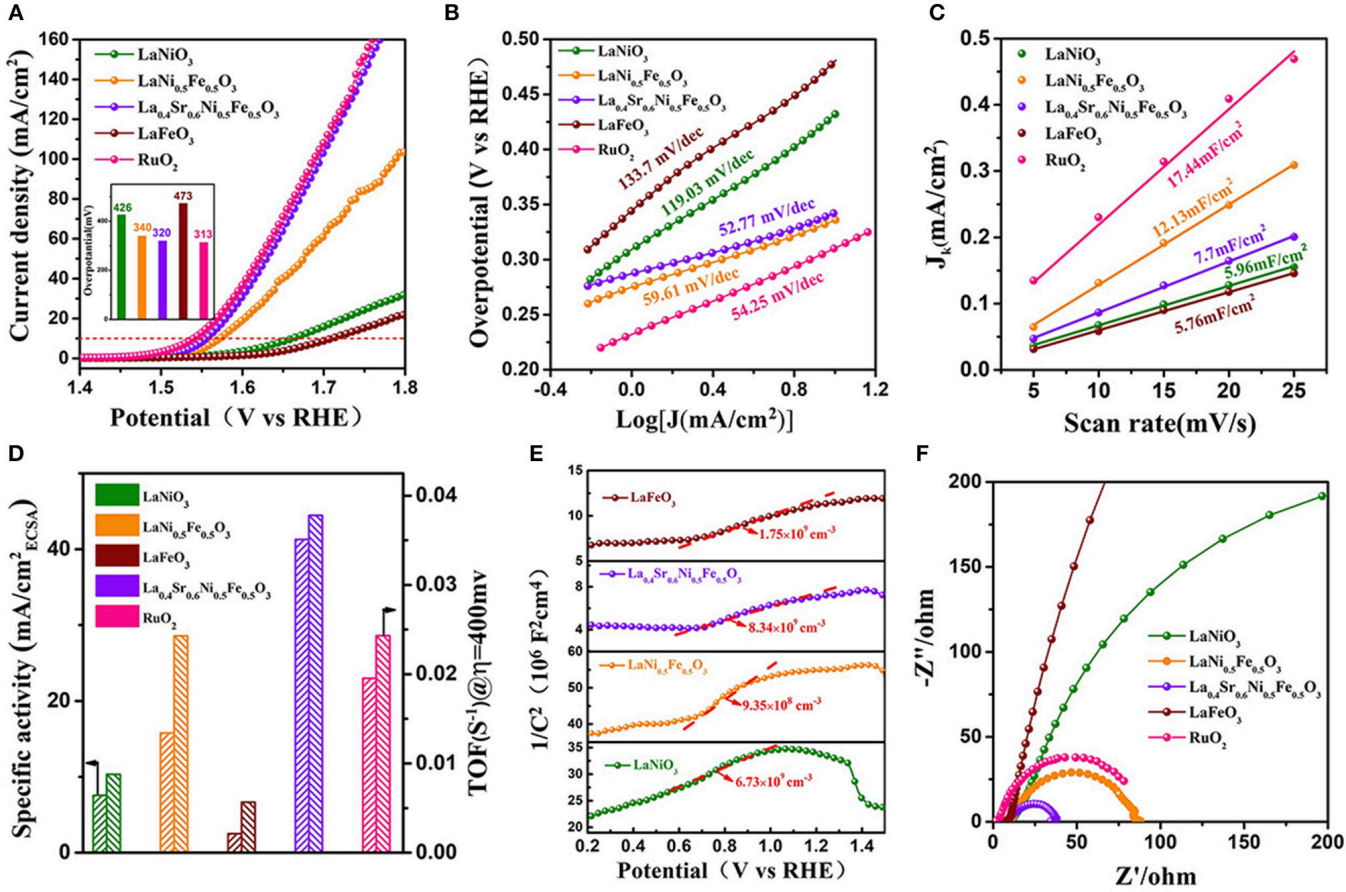
**(A)** Linear sweeping voltammogram curves of various catalysts (LaNiO_3_, LaNi_0.5_Fe_0.5_O_3_, La_0.4_Sr_0.6_Ni_0.5_Fe_0.5_O_3_, LaFeO_3_, and commercial RuO_2_) in 1.0 M KOH at a scan rate of 5 mV s^−1^; Inset compare the OER overpotential for the corresponding catalyst in **(A)** at J = 10 mA cm^−2^; **(B)** Tafel plots for the corresponding catalysts; **(C)** Plots of the current density vs. the scan rate to determine the double layer capacitance (*C*_*dl*_) of various catalysts; **(D)** Specific activity of various catalysts (OER current density @ 1.6 V vs. RHE normalized by electrochemical surface area) and TOF calculated at overpotential η = 400 mV; **(E)** Mott-Schottky plots for the corresponding various catalysts; **(F)** Electrochemical impedance spectra of various catalysts recorded at 1.58 V vs. RHE under the influence of an AC voltage of 5 mV.

The electrochemical performance of La_0.4_Sr_0.6_Ni_0.5_Fe_0.5_O_3_ was studied by a standard three-electrode system calibrated with a reversible hydrogen electrode (RHE) (Figure S5) in an O_2_-saturated 1 M KOH solution. For comparison, the electrochemical performance of LaNiO_3_, LaFeO_3_, LaNi_0.5_Fe_0.5_O_3_, and commercial RuO_2_ were also examined. From the linear scanning voltammogram (LSV) profile (Figure 2A, Figure S6), it can be seen that the OER overpotential of LaNi_0.5_Fe_0.5_O_3_ is 340 and 133 mV lower than the overpotentials of LaNiO_3_ (473 mV @ 10 mA cm^−2^). The overpotential further decreased to 320 mV for La_0.4_Sr_0.6_Ni_0.5_Fe_0.5_O_3_, almost the same to that of commercial RuO_2_. These results demonstrate the B-site Fe doping is the main reason for the overpotential decrease, and the A-site Sr doping could further optimize the performance. The OER kinetics of the catalyst were also evaluated by the tafel curves. La_0.4_Sr_0.6_Ni_0.5_Fe_0.5_O_3_ possesses a small tafel slope (52.77 mV dec^−1^) (Figure 2B), even lower than that of RuO_2_ (54.25 mV dec^−1^). Then the electrochemical active surface area (ECSA) was measured to provide information on the density of active sites (Chen et al., 2016) (Figures S7, S8). As shown in Figure 2C, the ECSA value of La_0.4_Sr_0.6_Ni_0.5_Fe_0.5_O_3_ (12.13 mF/cm^2^) was smaller than that of RuO_2_ (17.4 mF/cm^2^), which represented that the exposed high active sites of La_0.4_Sr_0.6_Ni_0.5_Fe_0.5_O_3_ is less than RuO_2_ in the OER progress. While the specific activity of La_0.4_Sr_0.6_Ni_0.5_Fe_0.5_O_3_ was calculated by normalizing the OER current at 1.6 V vs. RHE related to the corresponding ECSA, is much superior than that of LaNi_0.5_Fe_0.5_O_3_ and RuO_2_ (Figure 2D and Table S2), indicating the intrinsic catalytic activity of La_0.4_Sr_0.6_Ni_0.5_Fe_0.5_O_3_ is higher than RuO_2_. In addition, La_0.4_Sr_0.6_Ni_0.5_Fe_0.5_O_3_ exhibits an exceedingly high turnover frequency (TOF), (0.0378 s^−1^), 2.4 times larger than that of RuO_2_ (0.016 s^−1^) (Gao et al., 2018) (Figure 2D) further proving its excellent intrinsic activity (see detailed calculations in the Supporting Information). It can be seen from cyclic voltammetry curves (Figure S9) that the peak current density of the oxidation peak [within a certain margin of error, Ni^2+^/Ni^3+^ (1.39~1.45 V vs. RHE)] of La_0.4_Sr_0.6_Ni_0.5_Fe_0.5_O_3_ is higher than LaNiO_3_, LaFeO_3_ and LaNi_0.5_Fe_0.5_O_3_, indicating that La_0.4_Sr_0.6_Ni_0.5_Fe_0.5_O_3_ has the highest OER intrinsic catalytic activity (Vij et al., 2017). The ring test results (Figure S10) show that the FE of the OER is determined by the rotating ring disk electrode (RRDE), and the high FE value of perovskite La_0.4_Sr_0.6_Ni_0.5_Fe_0.5_O_3_ (97.1%) indicates that the observed oxidation current is completely originates from water oxidation rather than other side reactions.

It is well-known that the conductivity of catalyst is another key factor in the improvement of OER performance (Zhang et al., 2018). The carrier concentration (*Na*) of LaNiO_3_, LaNi_0.5_Fe_0.5_O_3_, La_0.4_Sr_0.6_Ni_0.5_Fe_0.5_O_3_, and LaFeO_3_ were estimated from the slope MS plots (Figure 2E, see detailed calculations in the Supporting Information). The calculated results show that the *Na* of La_0.4_Sr_0.6_Ni_0.5_Fe_0.5_O_3_ (8.34 10^9^ cm^−3^) (Figure S11) is higher than LaNiO_3_ (6.73 10^9^ cm^−3^), LaNi_0.5_Fe_0.5_O_3_ (9.35 10^8^ cm^−3^), and LaFeO_3_ (1.75 10^9^ cm^−3^), indicating that La_0.4_Sr_0.6_Ni_0.5_Fe_0.5_O_3_ has the highest electronic conductivity, which was also proved by the EIS test (Figure 2F).

**Figure 3:**
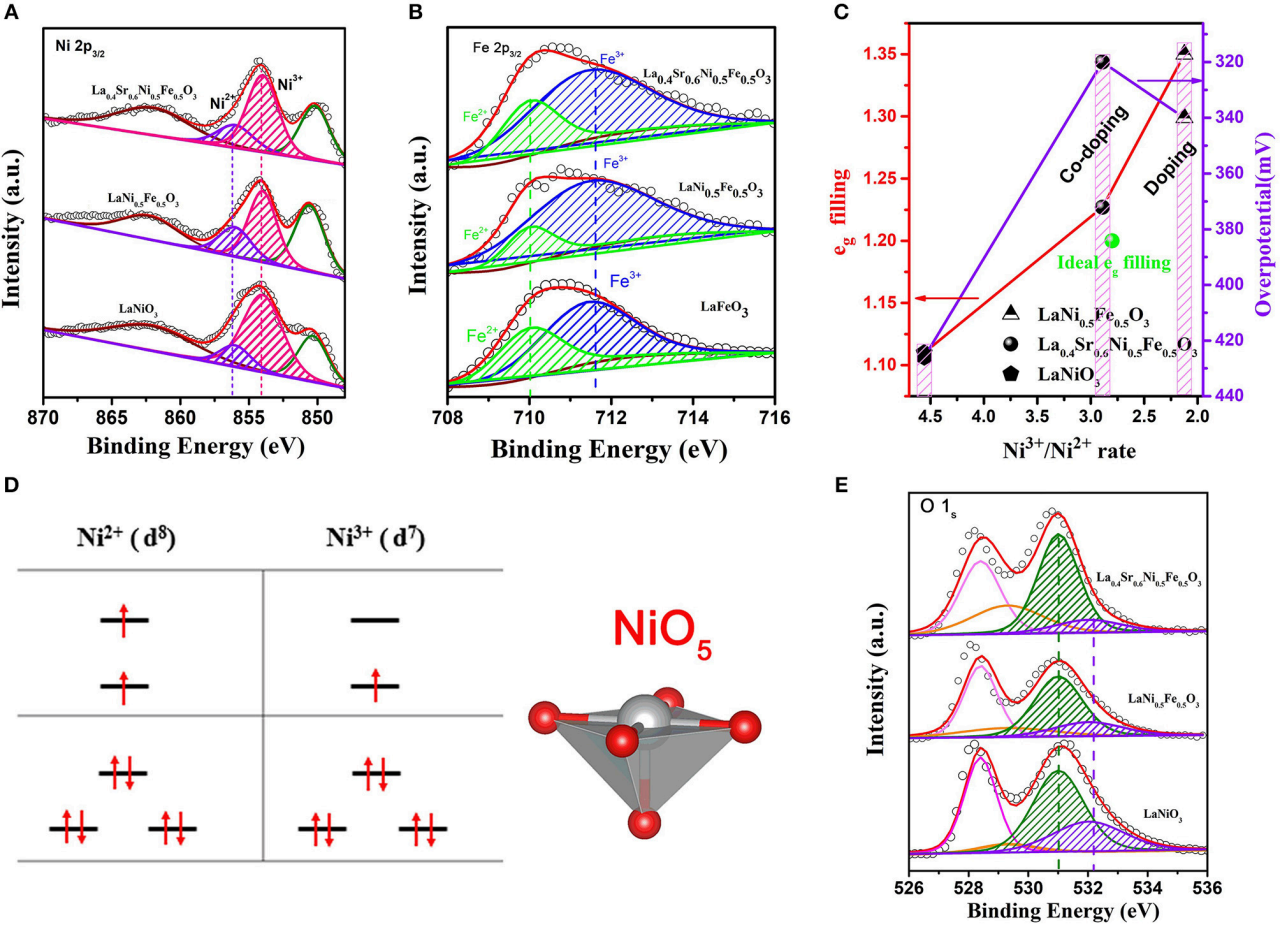
**(A)** Ni: 2p_3/2_, **(B)** Fe 2p_3/2_, and **(E)** O 1s spectra of LaNiO_3_, LaNi_0.5_Fe_0.5_O_3_, La_0.4_Sr_0.6_Ni_0.5_Fe_0.5_O_3_, and LaFeO_3_ perovskite oxides; The *e*_*g*_ orbital filling of perovskites. **(C)** *e*_*g*_ filling and OER activity as a function of Ni^3+^/Ni^2+^ rate; The green dot only represents the ideal *e*_*g*_ filling; **(D)** Electronic configuration and relevant metal orbitals of Ni^2+^ and Ni^3+^ for a NiO_5_ configuration.

To originate the high OER activity, X-ray photoelectron spectroscopy (XPS) of La_0.4_Sr_0.6_Ni_0.5_Fe_0.5_O_3_ were performed as shown in Figure S12. For comparison, XPS spectrum of LaNiO_3_, LaNi_0.5_Fe_0.5_O_3_ and LaFeO_3_ were also studied. In order to accurately obtain the valence and content of Ni on the perovskite catalyst surface, we then fitted the La:3d_3/2_ XPS spectrum at high binding energy and subtracted it (La: 3d_3/2_ and Ni: 2p_3/2_ XPS spectra overlap under high binding energy). The peaks at 854 and 856 eV in spectrum of Figure 3A are typical peaks of Ni^2+^ and Ni^3+^ (Yin et al., 2017), respectively. Based on the calculation of XPS peak area intensity (Table S1a), the ratio of Ni^3+^/Ni^2+^ for LaNiO_3_ is 4.559. After Fe doping, the ratio dramatically decreased to 2.123. While the ratio returned to 2.891 after Sr and Fe co-doping. Therefore, we can conclude that the ratio change of Ni^3+^/Ni^2+^ is mainly induced by the doping of Fe element, and the reason could be attributed to the existence of both Fe^2+^ and Fe^3+^ in LaNi_0.5_Fe_0.5_O_3_, and La_0.4_Sr_0.6_Ni_0.5_Fe_0.5_O_3_ (Figure 3B and Table S1b). In addition, the Sr doping could compensate the ratio decrease of Ni^3+^/Ni^2+^ induced by Fe doping. These different ratios of Ni^3+^/Ni^2+^ led to various average valences of Ni in different samples, i.e., 2.89, 2.65, and 2.773 for LaNiO_3_, LaNi_0.5_Fe_0.5_O_3_, and La_0.4_Sr_0.6_Ni_0.5_Fe_0.5_O_3_, respectively (Zhu et al., 2015b; Ge et al., 2016). It has been proved that the valence state of B-site metal ions on the catalyst surface are conducive to optimize the *e*_*g*_ (Seitz et al., 2016; Li et al., 2017; Vij et al., 2017), due to the electron number of M^x+^ (M = Ni, Co) in anti-bonding orbital (*e*_*g*_) is different (for Ni ions, Ni^3+^($t_{2g}^{6}$$e_{g}^{1}$), Ni^2+^($t_{2g}^{6}$$e_{g}^{2}$)) (Figure 3D) (Jin et al., 2011; Suntivich et al., 2011; Hwang et al., 2017). Therefore, the *e*_*g*_ filling is 1.1, 1.35, and 1.23 for LaNiO_3_, LaNi_0.5_Fe_0.5_O_3_, and La_0.4_Sr_0.6_Ni_0.5_Fe_0.5_O_3_, respectively, indicating that the *e*_*g*_ filling of Ni in LaNiO_3_ can be effectively optimized to an ideal value of 1.2 (green dot) by adjusting the ratio of Ni^3+^ and Ni^2+^ through the Sr and Fe co-doping (Figure 3C) (Hong et al., 2015). The M-T (Magnetizations Temperature-dependent) test also shows that La's *e*_*g*_ filling is close to 1.25 (Figure S13).

The optimal *e*_*g*_ filling resulted in a moderate adsorption of OH^−^ on the B-site metal atoms of the perovskite catalyst to form OH^*^ (Xu et al., 2016b), which is seen as the rate limiting step for OER of perovskite in alkaline solution, and thus leading to a high OER performance (Jin et al., 2011; Suntivich et al., 2011). As shown in Figure 3D, the exposed Ni sites have the coordination environment NiO_5_, with the apical (vertical) oxygen removed, which is benefit for the adsorption of OH^−8^. This could be proved by the XPS spectra of O 1s (Figure 3E). Upon deconvolution, the two peaks at low binding energy are assigned to the lattice oxygen combinate with La (528.6 eV) and Ni (529.3 eV), respectively. While the two peaks at high binding energy are assigned to the O atoms in the adsorbed OH^−^ on La (531.01 eV, La-OH) and Ni (532.02 eV, Ni-OH) (Zhang et al., 2015; Ge et al., 2016). Compared with other perovskite, the peaks strength of Ni-OH in La_0.4_Sr_0.6_Ni_0.5_Fe_0.5_O_3_ are moderate, indicating the appropriate OH^−^ adsorption on the surface of the catalyst (Suntivich et al., 2011; Hwang et al., 2017). Based on the above analysis results, La_0.4_Sr_0.6_Ni_0.5_Fe_0.5_O_3_ is a promising catalyst for OER in alkaline solution due to its appropriate OH^−^ adsorption.

**Figure 4:**
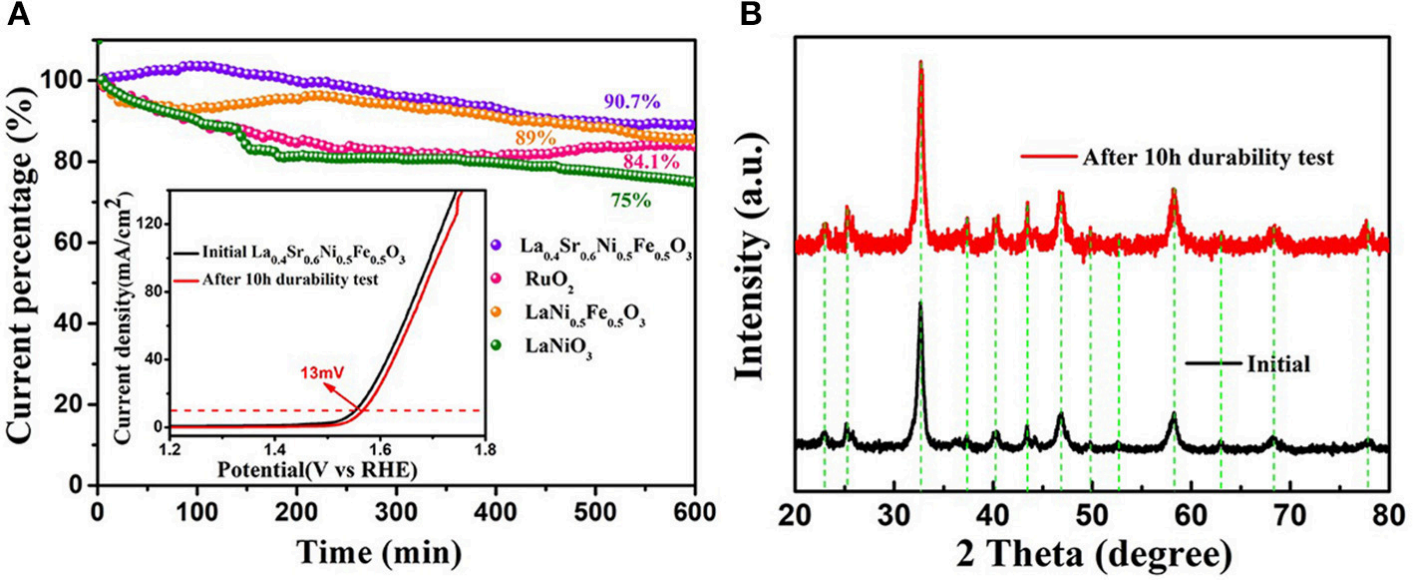
**(A)** Chronoamperometric response of La_0.4_Sr_0.6_Ni_05_Fe_0.5_O_3_, commercial RuO_2_, LaNi_0.5_Fe_0.5_O_3_ and LaNiO_3_, where inset shows the LSV curves of La_0.4_Sr_0.6_Ni_05_Fe_0.5_O_3_ before and after 10 h durability test. **(B)** The XRD patterns of La_0.4_Sr_0.6_Ni_0.5_Fe_0.5_O_3_ before and after 10 h durability test.

In addition to the excellent electrochemical activity, it is also required for catalysts to exhibit high stability in 1 M KOH in various engineering applications (Zhou et al., 2015). In the chronoamperometry test, compared with the LaNi_0.5_Fe_0.5_O_3_, LaNiO_3_, and commercial RuO_2_, the current density of La_0.4_Sr_0.6_Ni_0.5_Fe_0.5_O_3_ decreased only 9.3% after 10 h of continuous polarization (Figure 4A), which is comparable with the electrochemical performance of commercial RuO_2_. Moreover, the overpotential (@10 mA cm^−2^) increases only 13 mV, demonstrating the high durability of La_0.4_Sr_0.6_Ni_0.5_Fe_0.5_O_3_ (illustration of Figure 4A). Compared with the original La_0.4_Sr_0.6_Ni_0.5_Fe_0.5_O_3_, no heterogenic peak in XRD spectrum emerged after 10 h electrochemistry testing, demonstrating the high stability of the structure of the La_0.4_Sr_0.6_Ni_0.5_Fe_0.5_O_3_ (Figure 4B).

## Conclusion

In summary, the perovskite oxide La_0.4_Sr_0.6_Ni_0.5_Fe_0.5_O_3_ with an average size around 25 nm is obtained by a simple co-doping method, which exhibits a high OER performance in an alkaline solution (Table S3). Electrochemical test results indicate that the OER activity can be enhanced by changing the ratio of Ni^3+^/Ni^2+^ and optimizing its *e*_*g*_ filling, as well as the OH adsorption. Such significant intrinsic OER activity may result from the synergistic effect of the co-doping of Sr and Fe. Furthermore, the chronoamperometry test of La_0.4_Sr_0.6_Ni_0.5_Fe_0.5_O_3_ also demonstrated 9.3% decay for OER within 10 h. We tested the SEM of La_0.4_Sr_0.6_Ni_0.5_Fe_0.5_O_3_ after OER cycles test. Comparing the morphology of La_0.4_Sr_0.6_Ni_0.5_Fe_0.5_O_3_ before the test, it can be seen that the morphology of La_0.4_Sr_0.6_Ni_0.5_Fe_0.5_O_3_ did not change much after 10 h durability test while the particles had a certain agglomeration after the test (Figure S14). The electrochemical results indicate that the synthesized perovskite oxide La_0.4_Sr_0.6_Ni_0.5_Fe_0.5_O_3_ may become a promising catalyst in metal-air batteries and solar fuel applications.

## Data Availability

The raw data supporting the conclusions of this manuscript will be made available by the authors, without undue reservation, to any qualified researcher.

## Author Contributions

HL and ZL designed the project. QG performed the experiment under the direction of ZL. QG and XL performed the experimental data analysis. QG, HL, XL, ZL, XY, LL, YL, HW, and XZ developed the formation mechanism. QG, HL, and XL wrote the paper. All authors contributed to discussion of the results.

### Conflict of Interest Statement

The authors declare that the research was conducted in the absence of any commercial or financial relationships that could be construed as a potential conflict of interest.
