# Supplementary material for: Sr, Fe Co-doped Perovskite Oxides With High Performance for Oxygen Evolution Reaction
Source: Front Chem. 2019 Apr 24;7:224. doi: 10.3389/fchem.2019.00224 (PMC6491708; doi:10.3389/fchem.2019.00224)
Supplement: Supplementary file 1 [file Data_Sheet_1.doc]

***Supporting Information***

**Sr, Fe co-doped perovskite oxides with high performance for oxygen evolution reaction**

**Qiang Guo ^a^, Xiang Li ^a*^, Haifei Wei ^a^, Yi Liu ^a^, Lanlan Li ^a^, Xiaojing Yang ^a^, Xinghua Zhang ^a^, Hui Liu ^b*^, Zunming Lu ^a*^**

^a^ School of Materials Science and Engineering, Hebei University of Technology, Tianjin 300130, China

^b^ School of Materials Science and Engineering, Tianjin University, Tianjin, 300072, China

I. Calculation Details

***Calculation of turnover frequency (TOF)***

TOF was evaluated by following Equation ^[1]^:

$TOF=\frac{J\times A}{4\times F\times n}$, where *J* is the current density (A/cm^2^) at η=400 mV, *A* is the area of the glassy carbon electrode, *n* is the number of moles of catalyst active atoms on the electrode. *F* is the Faraday constant (96485 C mol^-1^).

***Calculation of carrier concentration (Na)***

The carrier concentration was evaluated by following Equation ^[2]^:

$\frac{dc^{-2}}{dv}=\frac{2}{q\varepsilon_{0}\varepsilon_{r}NaA^{2}}$***,*** where *ε_0_* is the dielectric constant of vacuum, *ε_r_* is the dielectric constant of the perovskite oxide, *q* is the charge of the electrons, *A* is the specific surface area (BET) of the perovskite oxide, *Na* is the carrier concentration.

***Calculation of e_g_ filling number for Ni^3+^ in perovskite***

The total effective magnetic moments (*µ*eff) for perovskite samples could be evaluated by using the Equation (1), according to the Langevin theory ^[3]^，

$\mu_{\text{eff}}=\sqrt{8C\mu_{B}}$ (1)

Where *C* is Curie constant and obtained from the fittings on the susceptibility (χ= *M*/*H*) above the paramagnetic transition temperatures by a Curie-Weiss law χ=*C* ⁄(*T*-*Θ*), where *Θ* is CurieWeiss temperature. *μ_eff_* of perovskite includes contributions from both magnetic ions, i.e., Ni^3+^ and Fe^3+^ and obeys Equation (2)

$\mu_{\text{eff}}^{2}=\mu_{\text{eff,Ni}^{2}}+\mu_{\text{Fe}}^{2}$ (2)

where µ_Fe_ is the magnetic moment of Fe^3+^.

$\mu_{\text{Fe}}^{2}=g^{2}\mu_{B}^{2}S(S+1)$ (3)

where the Lande factor *g*=2. Since Fe^3+^ has two possible spin state, the *S* value equals 1. Therefore, *µ*_Fe_=4.89*µ_B_.* Consequently, *µ*eff, Ni for Ni^3+^ in the perovskite samples could be derived from the measured *µ*eff by deducting the contribution of *µ*Fe based on Equation (1). For Ni ions, *µ_eff_* can also be calculated from the relationship:

(4)

where g is g factor, SLS(=1) and SHS(=1) are the S value, and VLS and VHS(=1- VLS) are the volume fractions for Ni^3+^ ions. Therefore, using above two values, the *e_g_* electron (x) can be further calculated by x= SLS*VLS +SHS*VHS. Based on the above calculations, we can calculate the *e_g_* filling of LaNiO_3_, LaNi_0.5_Fe_0.5_O_3_ and La_0.4_Sr_0.6_Ni_0.5_Fe_0.5_O_3_ is 1.073, 1.376 and 1.25 respectively.

II. Supplementary Results


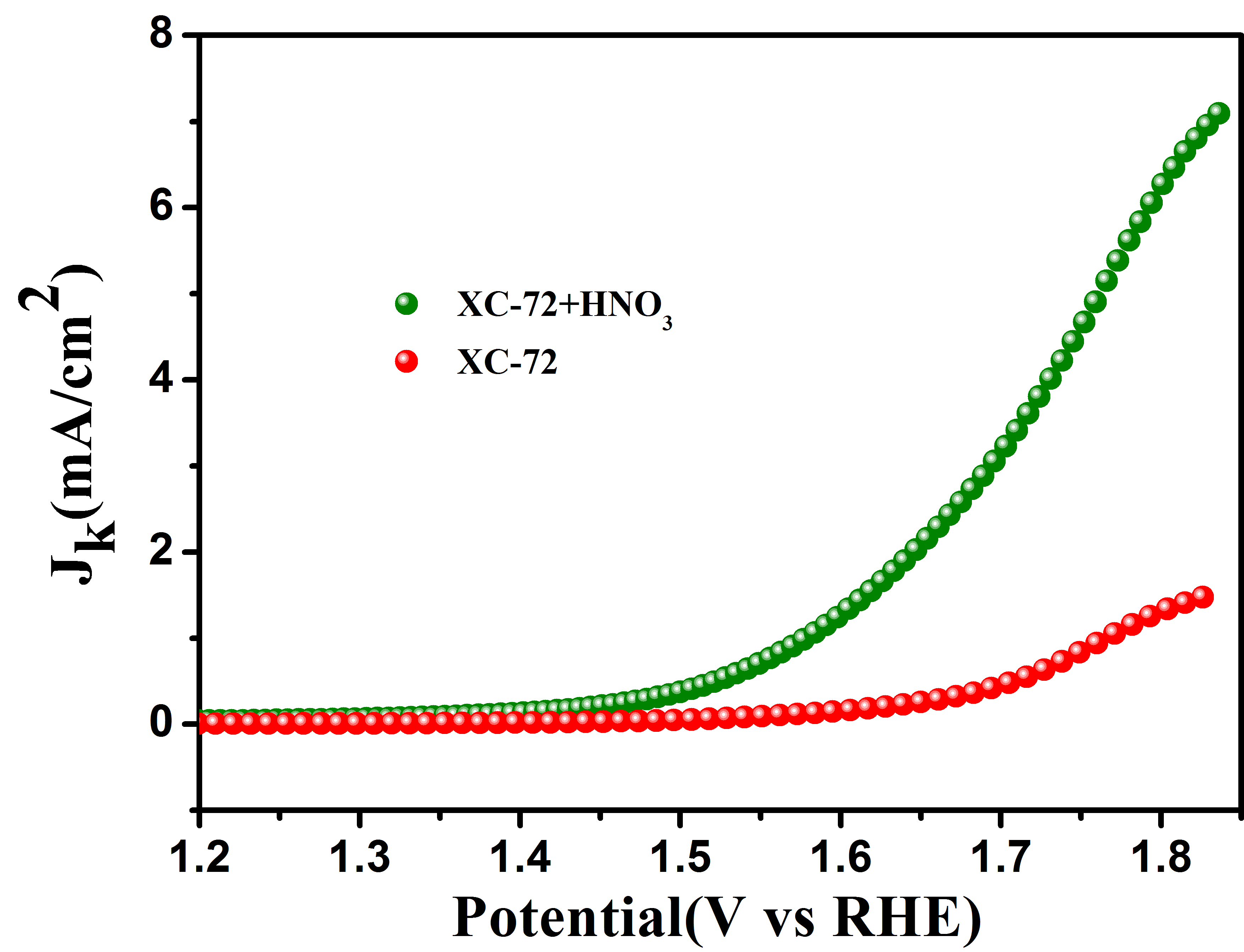


**Figure S1.** The Linear-sweep voltammograms (LSV) of XC-72 carbon black and nitric acid treated XC-72 carbon black in 1 M KOH solution.


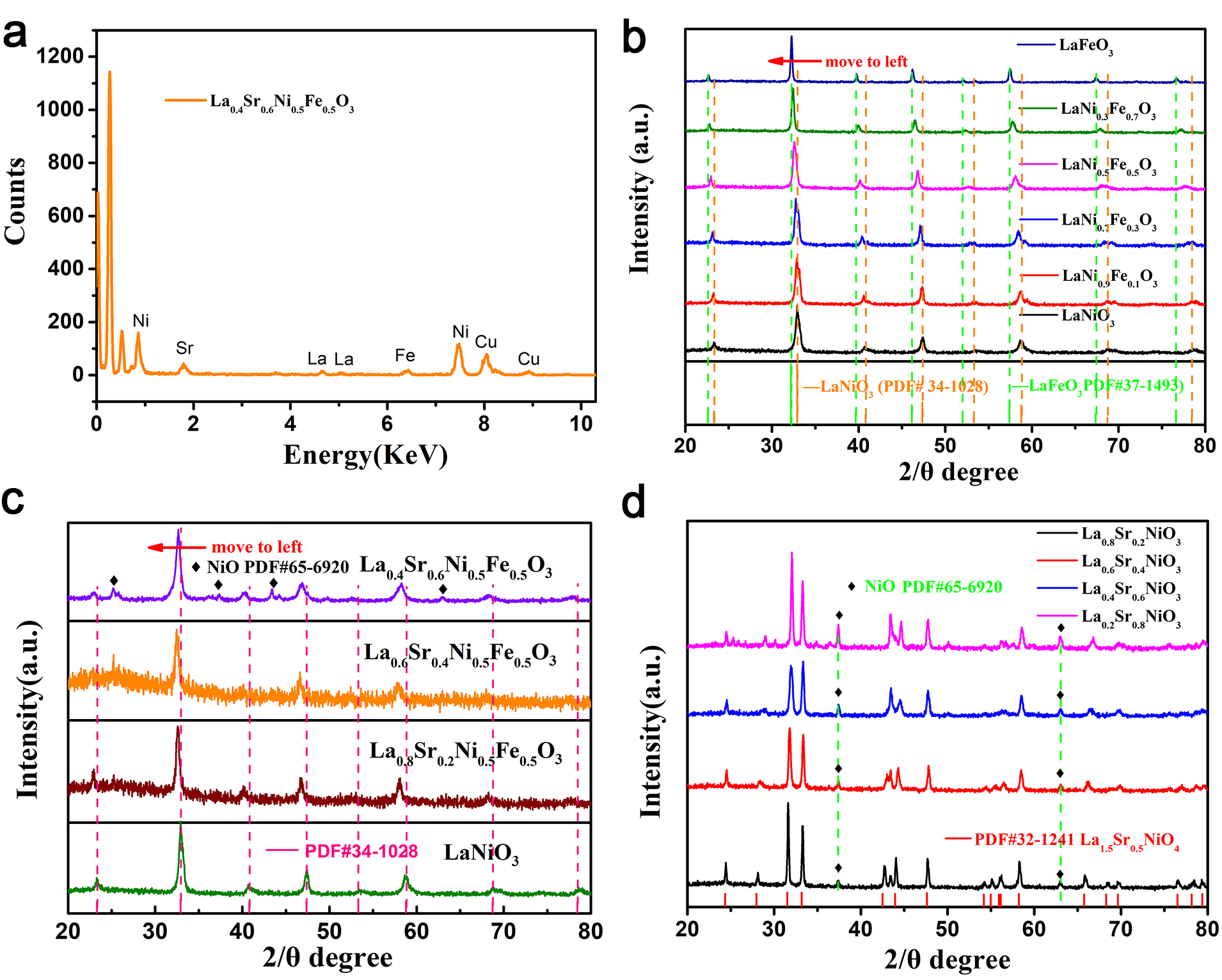


**Figure S2** a) The EDS patterns of La_0.4_Sr_0.6_Ni_0.5_Fe_0.5_O_3_ b) The XRD patterns of LaNi_1-x_Fe_x_O_3_ (x= 0.1, 0.3, 0.5, 0.7, 1) and c) La_1-y_SryNi_0.5_Fe_0.5_O_3_ (y= 0.2, 0.4, 0.6); and d) La_1-z_Sr_z_NiO_3_ (z= 0.2, 0.4, 0.6, 0.8).

**
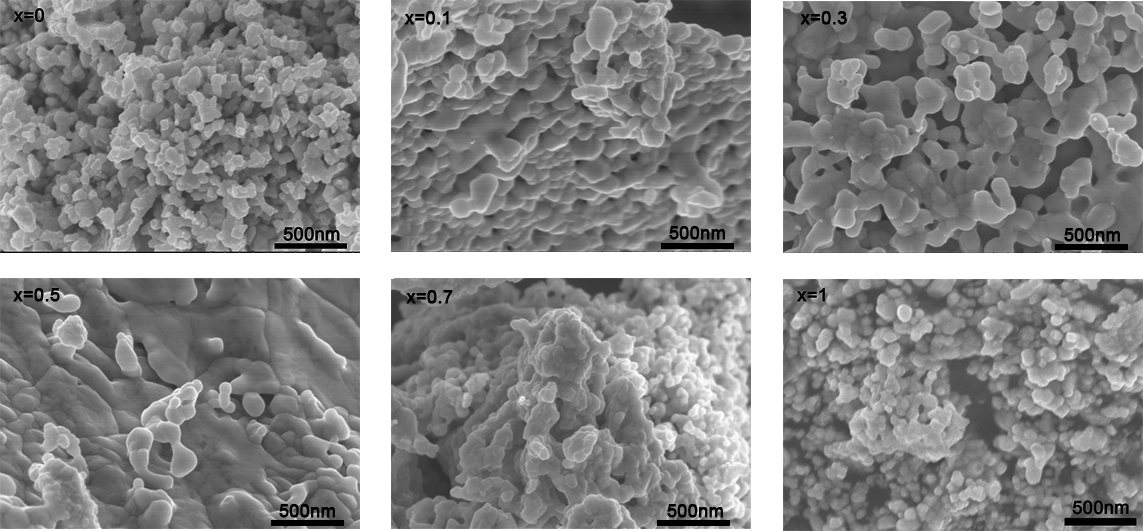
**

**Figure S3.** SEM images of perovskite LaNi_1-x_Fe_x_O_3_(x=0~1) oxides**.**


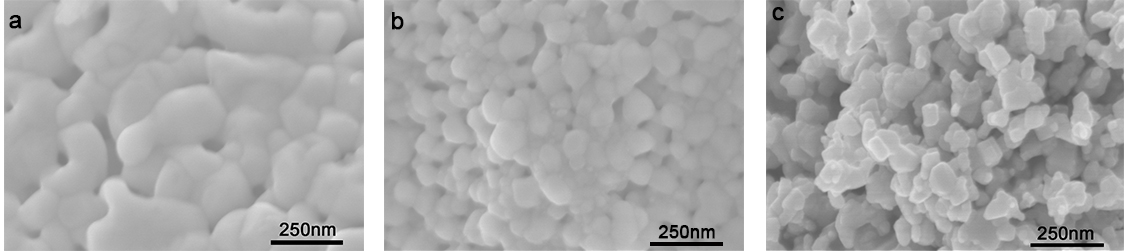


**Figure S4.** SEM images of spinel (a) La_0.8_Sr_0.2_Ni_0.5_Fe_0.5_O_3_, (b) La_0.6_Sr_0.4_Ni_0.5_Fe_0.5_O_3_, (c) La_0.4_Sr_0.6_Ni_0.5_Fe_0.5_O_3_ perovskite oxides.


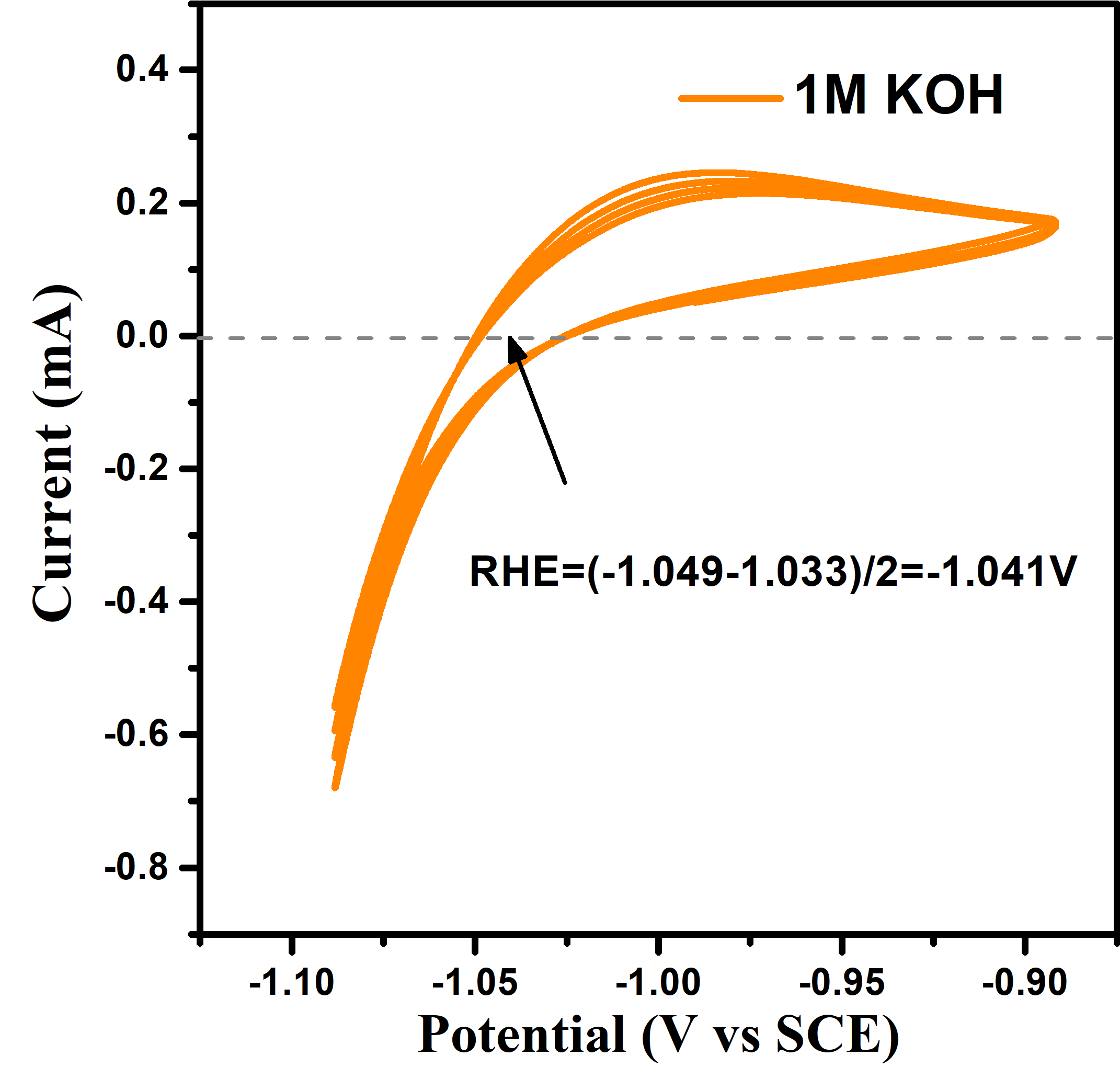


**Figure S5**. RHE calibration of the saturated calomel electrode (SCE).

**
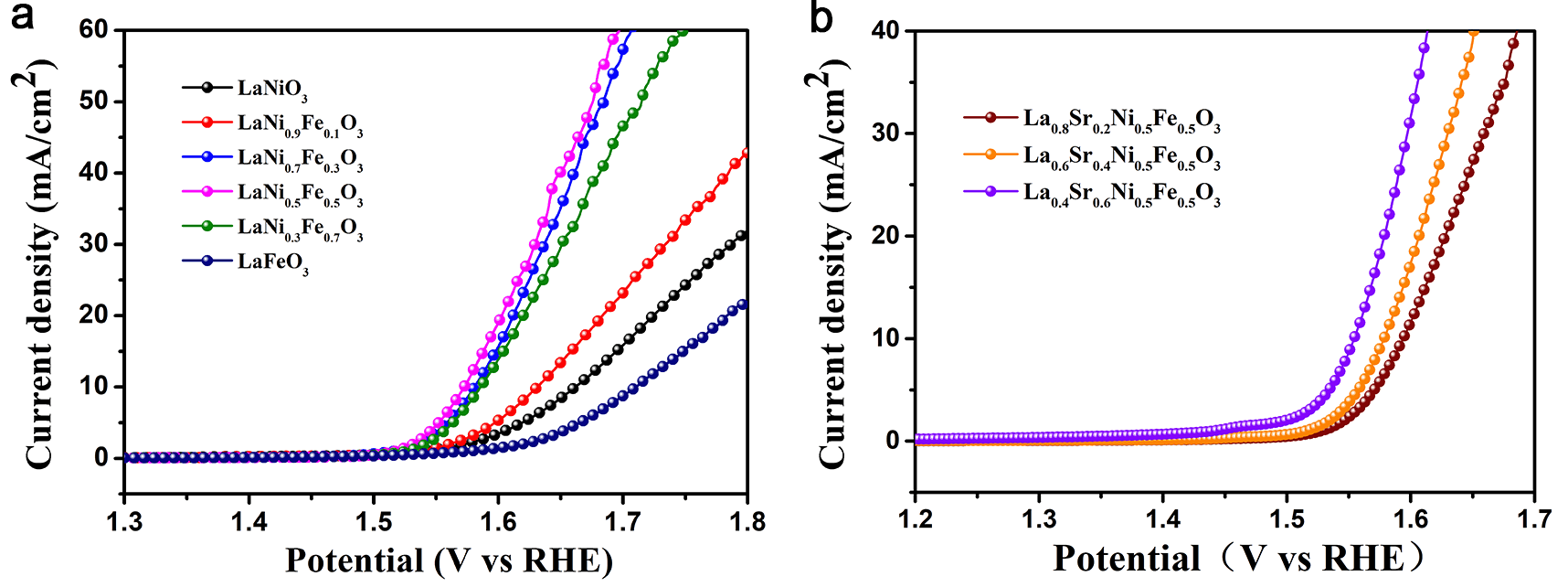
**

**Figure S6.** Electrocatalytic OER performance of a) LaNi_1-x_Fe_x_O3 (x=0.1,0.3,0.5,0.7,1) and b) La_1-y_Sr_y_Ni_0.5_Fe_0.5_O_3_ (y=0.2,0.4,0.6).


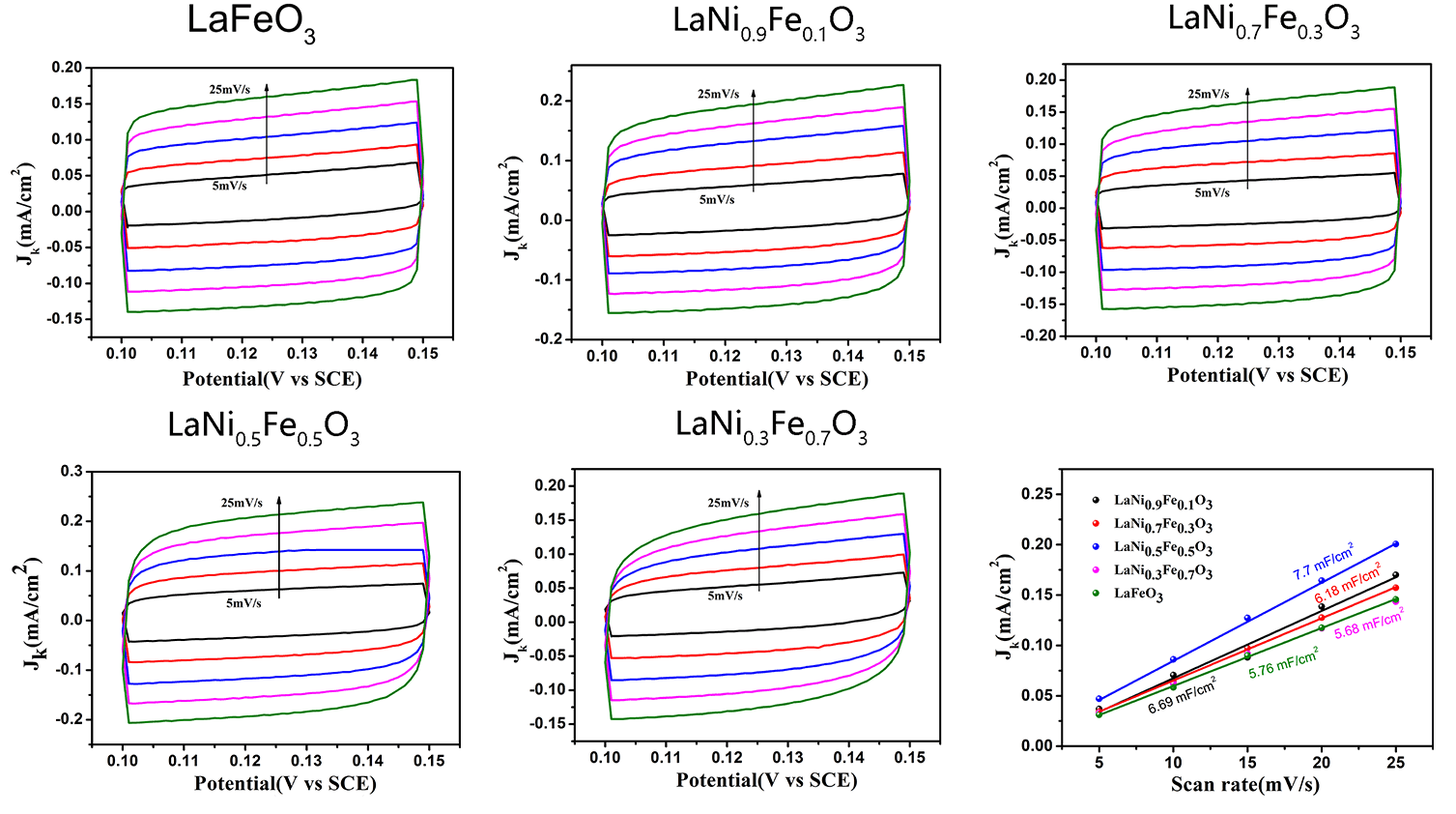


**Figure S7.** CV curves of LaFeO_3_, LaFe_0.9_Ni_0.1_O_3_, LaFe_0.7_Ni_0.3_O_3_, LaFe_0.5_Ni_0.5_O_3_, LaNi_0.3_Fe_0.7_O_3_ recorded in O_2_ saturated 1 M KOH solution at different scan rates. And Plots of the current density versus the scan rate to determine the double layer capacitance (*C*_dl_) of perovskite catalysts.

**
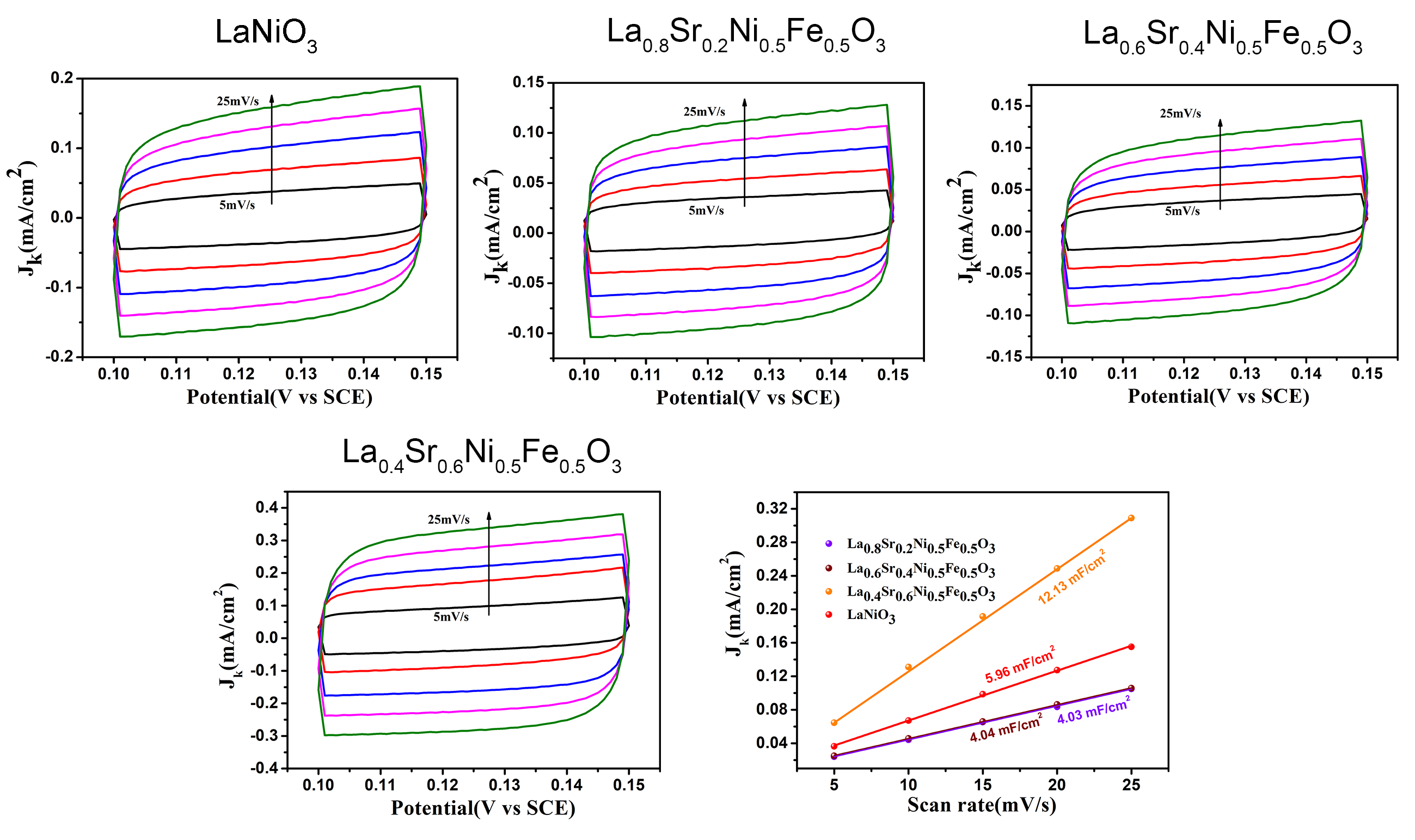
**

**Figure S8.** CV curves of La_0.8_Sr_0.2_Fe_0.9_Ni_0.1_O_3_, La_0.6_Sr_0.4_Fe_0.7_Ni_0.3_O_3_, La_0.4_Sr_0.6_Fe_0.5_Ni_0.5_O_3_ recorded in O_2_ saturated 1 M KOH solution at different scan rates. And Plots of the current density versus the scan rate to determine the double layer capacitance (*C*_dl_) of perovskite catalysts.


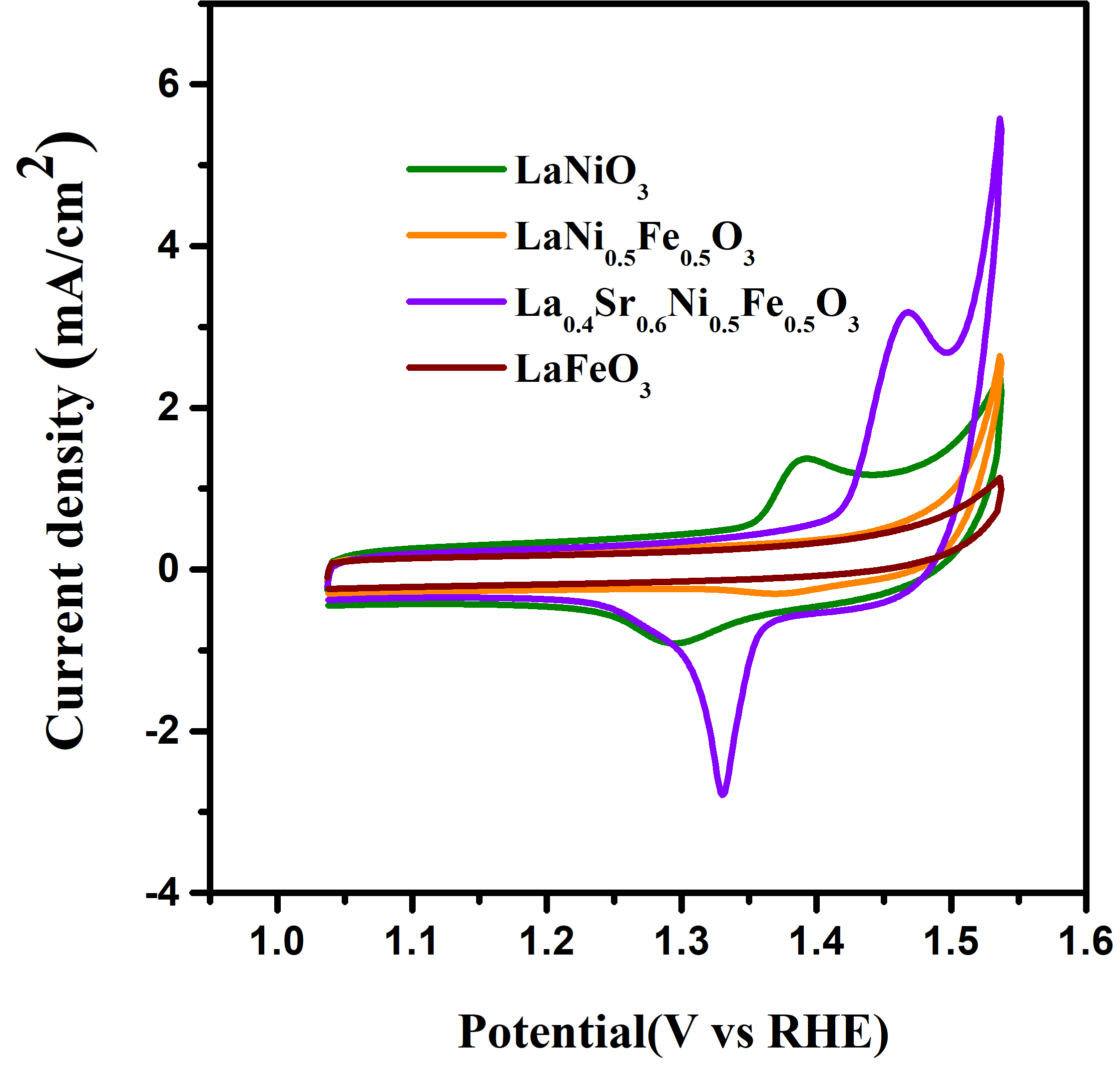


**Figure S9.** CV curves with a potential window at 0~0.5V vs SCE of perovskite catalysts.


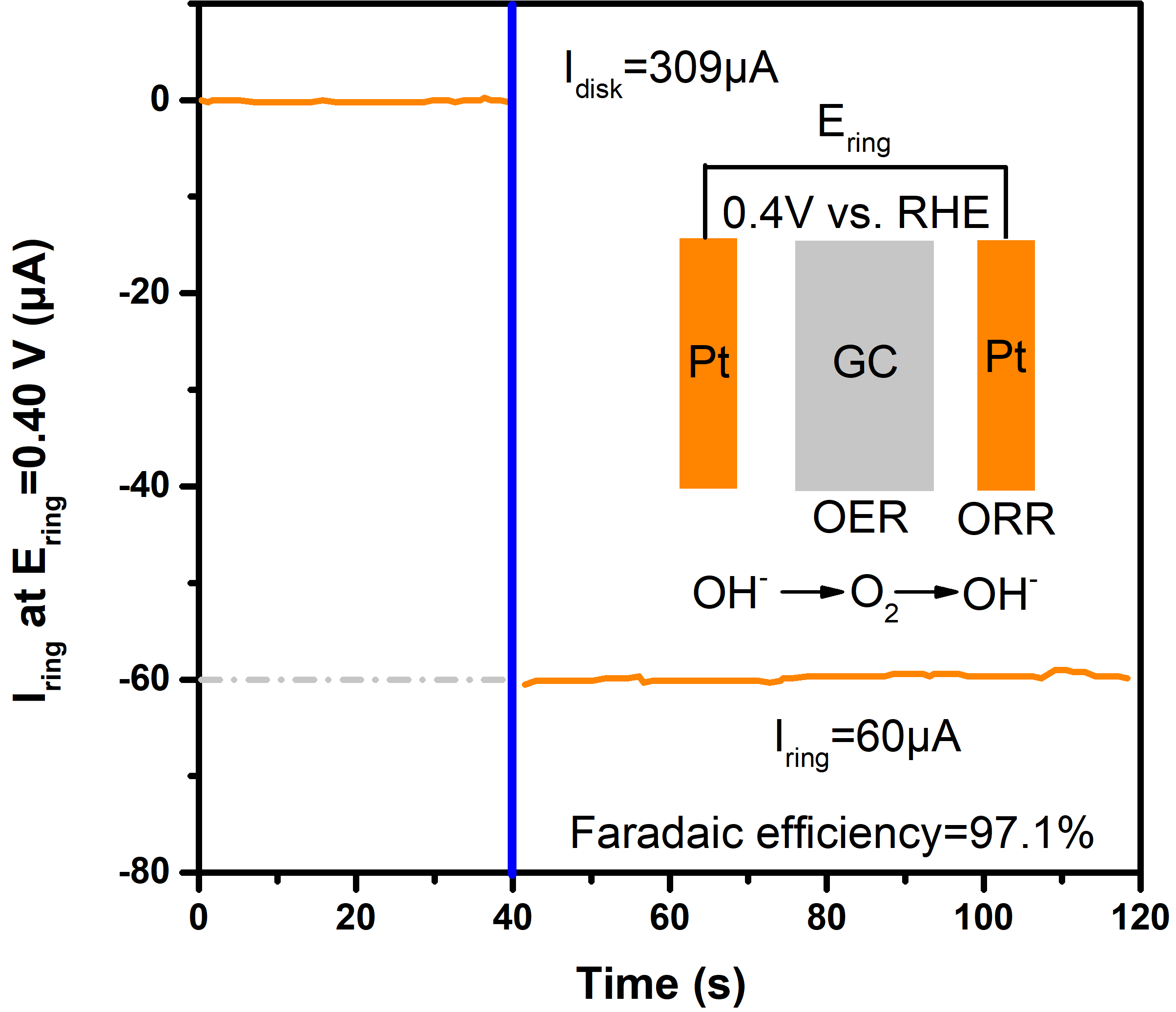


Figure S10. Ring current of perovskite La_0.4_Sr_0.6_Ni_0.5_Fe_0.5_O_3_ on an RRDE (1600 rpm) in N2-saturated 1 M KOH solution (ring potential 0.40 V).

**
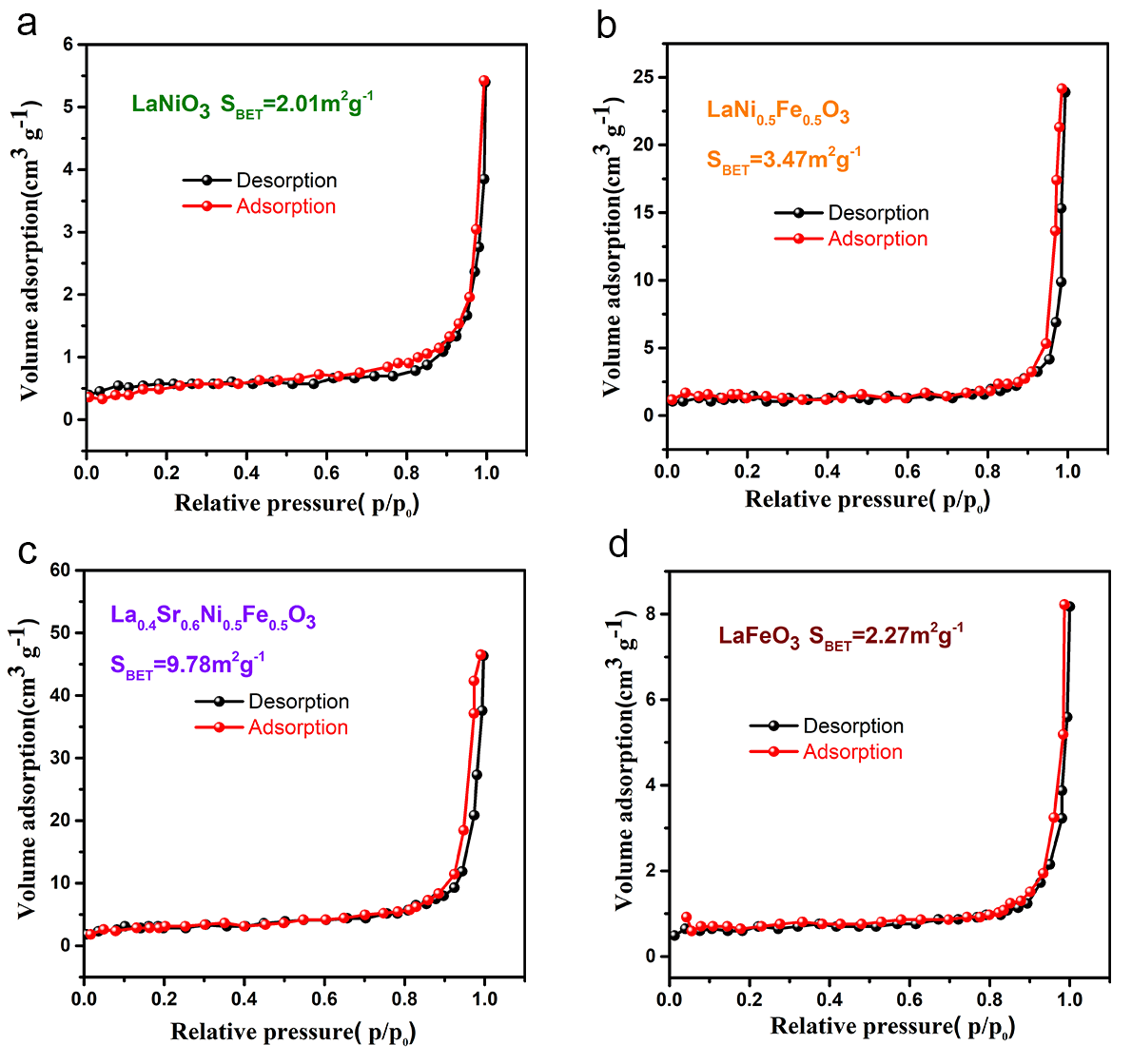
**

**Figure S11** Nitrogen adsorption/desorption isotherms of LaNiO_3_, LaNi_0.5_Fe_0.5_O_3_, La_0.4_Sr_0.6_Ni_0.8_Fe_0.2_O_3_, and LaFeO_3_. The BET surface areas of LaNiO_3_, LaNi_0.5_Fe_0.5_O_3_, La_0.4_Sr_0.6_Ni_0.8_Fe_0.2_O_3_ and LaFeO_3_ are calculated to be 2.01, 3.47, 9.78 and 2.27 m^2^ g^-1^, respectively.

**
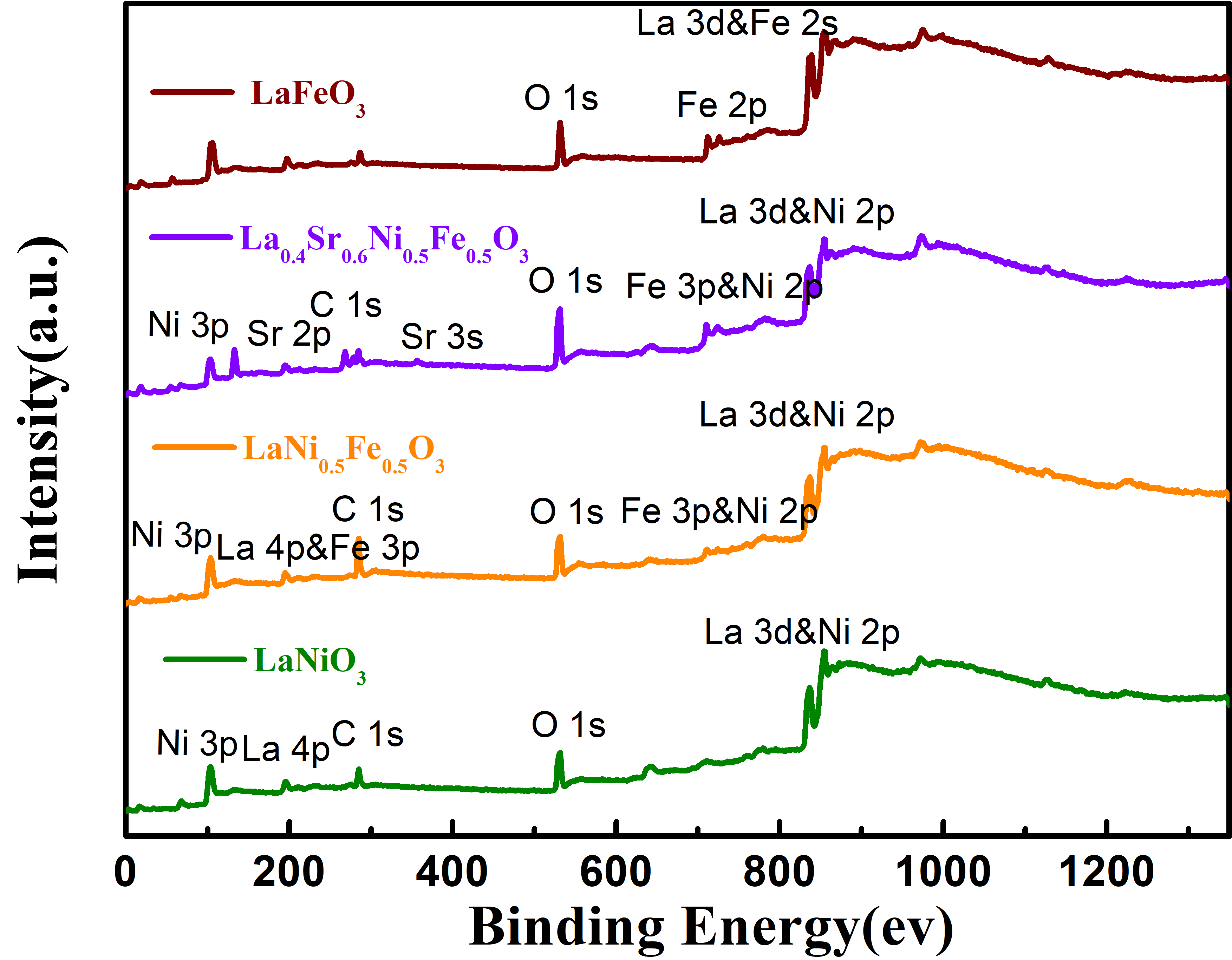
**

**Figure S12.** XPS survey spectra of as-prepared perovskite powders, LaNiO_3_, LaNi_0.5_Fe_0.5_O_3_

La_0.4_Sr_0.6_Ni_0.5_Fe_0.5_O_3_ and LaFeO_3_.


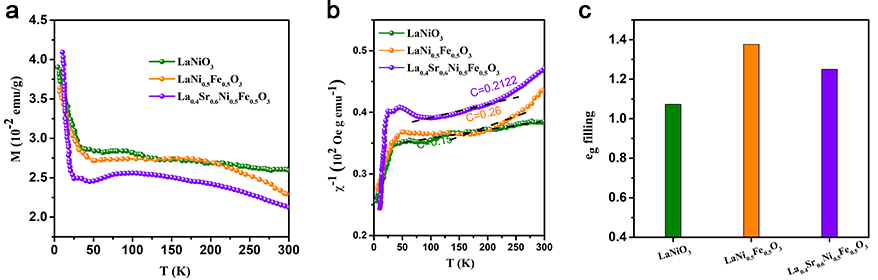


Figure S13 (a) The temperature dependence inverse susceptibilities for all the perovskite samples under H = 1 kOe. (b) *χ*-1-*T* curves and the dotted lines are the fitting results by a Curie–Weiss law. (c) The corresponding *e_g_* filling.


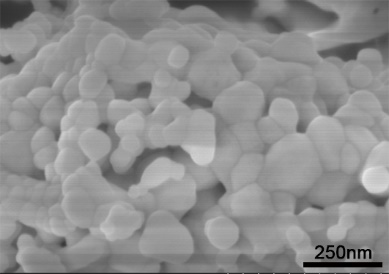


Figure S14**.** SEM image of the perovskite La_0.4_Sr_0.6_Ni_0.5_Fe_0.5_O_3_ after OER test.

**Table S1.** Relative peak areas of different binding energies fitted from XPS spectra, (a) Ni 2p_3/2_, (b) Fe 2p_3/2_, (c) O 1s

**a) Ni 2p_3/2_**

**Position (eV)**

| **Sample** | **Area** | | **Percentage (%)** | | **The average valence state of Ni** |
| --- | --- | --- | --- | --- | --- |
|  | **Ni^2+^（856eV）** | **Ni^3+^（854eV）** | **Ni^2+^ (%)** | **Ni^3+^ (%)** |  |
| LaNiO_3_ | 5047.165 | 23009.86 | **17.9885%** | 82.011% | **2.89** |
| LaNi_0.5_Fe_0.5_O_3_ | 4136.162 | 8782.19 | **32.0177%** | 67.9822% | **2.65** |
| La_0.4_Sr_0.6_Ni_0.5_Fe_0.5_O_3_ | 3720.92 | 10757.87 | **25.699%** | 74.301% | **2.773** |

**b) Fe 2p3/2**

**Position (eV)**

| **Sample** | **Area** | | **Percentage (%)** | |
| --- | --- | --- | --- | --- |
|  | **Fe^2+^（710.0eV）** | **Fe^3+^（711.4eV）** | **Fe^2+^ (%)** | **Fe^3+^ (%)** |
| LaFeO_3_ | 4640.223 | 2598.515 | **64.102%** | **35.898%** |
| LaNi_0.5_Fe_0.5_O_3_ | 511.455 | 2477.074 | **17.1139%** | **82.8861%** |
| La_0.4_Sr_0.6_Ni_0.5_Fe_0.5_O_3_ | 1779.54 | 4737.031 | **27.308%** | **72.692%** |

**c) O 1s**

| **Sample**  **Position (eV)** | **Area** | | | | **Percentage (%)** | |
| --- | --- | --- | --- | --- | --- | --- |
|  | **La-O（528.4eV）** | **Ni-O**  **（529.3eV）** | **La-O**  **（531.02eV）** | **Ni-O**  **(532.02eV)** | **La-O**  **（531.02eV）** | **Ni-O**  **(532.02eV)** |
| LaNiO_3_ | 7195.884 | 1136.731 | 9498.061 | 4986.991 | 41.627% | **21.856%** |
| LaNi_0.5_Fe_0.5_O_3_ | 5706.332 | 2048.274 | 6811.075 | 2349.022 | 40.267% | **13.887%** |
| La_0.4_Sr_0.6_Ni_0.5_Fe_0.5_O_3_ | 7768.443 | 5020.718 | 12526.63 | 2364.566 | 45.25% | **10.435%** |

**Table S2 Specific activity and TOF of different samples.**

| **Catalysts** | **Specific Activity**  **(mA cm^-2^_ECSA_)** | **TOF @ *η* = 400 mV**  **(s^-1^)** |
| --- | --- | --- |
| **LaNiO_3_** | 7.546 | 0.00879 |
| **LaNi_0.5_Fe_0.5_O_3_** | 15.77 | 0.0243 |
| **La_0.4_Sr_0.6_Ni_0.5_Fe_0.5_O_3_** | 41.28 | 0.0378 |
| **LaFeO_3_** | 2.501 | 0.005671 |
| **RuO_2_** | 22.04 | 0.016 |

**Table S3** Summary of the recently reported highly active perovskite-based OER catalysts in alkaline solution

| Catalyst | substrate | Electrolyte | Tafel slope (mV/dec) | η@10mA cm^-2^(mV) | Ref. |
| --- | --- | --- | --- | --- | --- |
| La_0.4_Sr_0.6_Ni_0.5_Fe_0.5_O_3_ | Glass Carbon | 1M KOH | 52.77 | 320 | This work |
| LaNi_0.85_Mg_0.15_O_3_ | Glass Carbon | 1M KOH | N.A.^[a]^ | 637 | J. Power Sources 2014 ^[4]^ |
| LaNi_0.8_Fe_0.2_O_3_ | Glass Carbon | 1M KOH | N.A. | 437 | J. Mater. Chem. A 2015 ^[5]^ |
| Ba_0.5_Sr_0.5_Co_0.8_Fe_0.2_O_3_ | Glass Carbon | 1M KOH | 65 | 350 | J. Hydrogen Energy 2016 ^[6]^ |
| La_0.5_Sr_0.5_Co_0.8_Fe_0.2_O_3_ | N.A. | 1M KOH | N.A. | 500 | Nano Energy 2014 ^[7]^ |
| La_0.58_Sr_0.4_Co_0.8_Fe_0.2_O_3_ | Glass Carbon | 1M KOH | N.A | 420 | Electrochim Acta 2016 ^[8]^ |
| (La_0.8_Sr_0.2_)_0.95_Mn_0.95_Ir_0.05_O_3 -δ_ | Glass Carbon | 0.1M KOH | 103 | 415 | ACS Appl. Mater. Interfaces 2017 ^[9]^ |
| LaNiO_3_ | Glass Carbon | 1M KOH | 80 | 430 | J. Phys.Chem. Lett. 2013 ^[10]^ |
| BaCo_0.7_Fe_0.2_Sn_0.1_O_3-δ_ | Glass Carbon | 0.1M KOH | 69 | 390 | Adv. Sci. 2016 ^[11]^ |
| SrNb_0.1_Co_0.7_Fe_0.2_O_3-δ_ | Glass Carbon | 0.1M KOH | 76 | 500 | Angew. Chem. Int. Ed. 2015^[12]^ |
| Ba_0.5_Sr_0.5_Co_0.8_Fe_0.2_O_3-δ_ | Glass Carbon | 0.1M KOH | N.A. | 500 | Adv. Mater. 2015 ^[13]^ |
| p-SnNiFe | N.A. | 0.1M KOH | 35 | 350 | Nat. Commun. 2017 ^[14]^ |
| NF-oLCFO-Ar | Nickel Foam | 0.1M KOH | 59 | 350 | Sci. Adv. 2016 ^[15]^ |
| La_0.5_Sr_0.5_Co_0.8_Fe_0.2_O_3_ | Glass Carbon | 0.1M KOH | 62.5 | 356 | Adv Mater Interfaces,2017 ^[16]^ |
| SrCo_0.9_5P_0.05_O_3-δ_ | Glass Carbon | 0.1M KOH | 84 | 495 | Adv Funct Mater,2016 ^[17]^ |
| NiO-(La_0.613_Ca_0.387_)_2_NiO_3.562_ | Glass Carbon | 0.1M KOH | 42 | 373 | Nano Energy, 2015 ^[18]^ |
| La_0.8_Sr_0.2_Mn_0.6_Ni_0.4_O_3_ | Glass Carbon | 0.1M KOH | N.A. | 607 | ACS Appl. Mater. Interfaces 2016 ^[19]^ |
| Ba_0.9_Co_0.5_Fe_0.4_Nb_0.1_O_3-δ_ | N.A. | 0.1M KOH | N.A. | 435 | Int. J. Hydrogen. Energy 2014 ^[20]^ |
| SrSc_0.025_Nb_0.075_Co_0.9_O_3-δ_ | Glass Carbon | 0.1M KOH | N.A. | 350 | Mater Horiz, 2015 ^[21]^ |
| SrNb_0.1_Co_0.7_Fe_0.2_O_3-δ_ | Polymer fibers | 0.1M KOH | 61 | 390 | Adv. Energy Mater., 2015 ^[22]^ |

[a]N.A.：not availiable

References

Z.W. Gao, T. Ma, X.M. Chen, H. Liu, L. Cui, [S.Z. Qiao](https://s2.sci-hub.org.cn/extdomains/scholar.google.com.hk/citations?user=B0bo5SUAAAAJ&hl=zh-TW&oi=sra), J. Yang, X.W. Du, Strongly coupled CoO nanoclusters/CoFe LDHs hybrid as a synergistic catalyst for electrochemical water oxidation, Small 14 (2018) 1800195.

T. Zhang, M.Y. Wu, D.Y. Yan, J. Mao, H. Liu, W.B. Hu, X.W .Du, T. Ling, S.Z. Qiao, Engineering oxygen vacancy on NiO nanorod arrays for alkaline hydrogen evolution, Nano Energy 43 (2018) 103-109.

S. Zhou, X. B. Miao, X. Zhao, C. Ma, Y. Qiu, Z. P. Hu, J. Y. Zhao, L. Shi, J. Zeng, Engineering electrocatalytic activity in nanosized perovskite cobaltite through surface spin-state transition. Nature communications 7 (2016) 11510.

Z.Z. Du, [P. Yang](https://s2.sci-hub.org.cn/extdomains/scholar.google.com.hk/citations?user=ID4eGCcAAAAJ&hl=zh-TW&oi=sra), L. Wang, Y.H. Lu, J.B. Goodenough, J. Zhang, D.W. Zhang, Electrocatalytic performances of LaNi_1−x_Mg_x_O_3_ perovskite oxides as bi-functional catalysts for lithium air batteries, Journal of Power Sources 265 (2014) 91-96.

D.W. Zhang, Y.F. Song, Z.Z. Du, L. Wang, [Y.T Li](https://s2.sci-hub.org.cn/extdomains/scholar.google.com.hk/citations?user=fqR_UVYAAAAJ&hl=zh-TW&oi=sra), J.B. Goodenough, Active LaNi_1-x_Fe_x_O_3_ bifunctional catalysts for air cathodes in alkaline media, Journal of Materials Chemistry A 18 (2015) 9421-9426.

[J. Wang](https://s2.sci-hub.org.cn/extdomains/scholar.google.com.hk/citations?user=x5G8KKYAAAAJ&hl=zh-TW&oi=sra), H. Zhao, [Y. Gao](https://s2.sci-hub.org.cn/extdomains/scholar.google.com.hk/citations?user=3qEa8JsAAAAJ&hl=zh-TW&oi=sra), [D.J. Chen](https://s2.sci-hub.org.cn/extdomains/scholar.google.com.hk/citations?user=WLAAftMAAAAJ&hl=zh-TW&oi=sra), [C. Chen](https://s2.sci-hub.org.cn/extdomains/scholar.google.com.hk/citations?user=3TpB6VQAAAAJ&hl=zh-TW&oi=sra), M. Saccoccio, F. Ciucci, Ba_0.5_Sr_0.5_Co_0.8_Fe_0.2_O_3−δ_, on N-doped mesoporous carbon derived from organic waste as a bi-functional oxygen catalyst, International Journal of Hydrogen Energy 41 (2016) 10744-10754.

H.W. Park, [D.U. Lee](https://s2.sci-hub.org.cn/extdomains/scholar.google.com.hk/citations?user=TAR8xsAAAAAJ&hl=zh-TW&oi=sra), [P. Zamani](https://s2.sci-hub.org.cn/extdomains/scholar.google.com.hk/citations?user=gwOhCOsAAAAJ&hl=zh-TW&oi=sra), [M.H. Seo](https://s2.sci-hub.org.cn/extdomains/scholar.google.com.hk/citations?user=CzcGMQUAAAAJ&hl=zh-TW&oi=sra), [L.F. Nazar](https://s2.sci-hub.org.cn/extdomains/scholar.google.com.hk/citations?user=y6mKPBkAAAAJ&hl=zh-TW&oi=sra), Z.W. Chen, Electrospun porous nanorod perovskite oxide/nitrogen-doped graphene composite as a bi-functional catalyst for metal air batteries, Nano Energy 10 (2014) 192-200.

K. Elumeeva, [J. Masa](https://s2.sci-hub.org.cn/extdomains/scholar.google.com.hk/citations?user=3O2I5BcAAAAJ&hl=zh-TW&oi=sra), J. Sierau, F. Tietz, [M. Muhler](https://s2.sci-hub.org.cn/extdomains/scholar.google.com.hk/citations?user=L_yJKOEAAAAJ&hl=zh-TW&oi=sra), W. Schuhmann, Perovskite-based bifunctional electrocatalysts for oxygen evolution and oxygen reduction in alkaline electrolytes, Electrochimica Acta 208 (2016) 25-32.

L. Yan, [Y. Lin](https://s2.sci-hub.org.cn/extdomains/scholar.google.com.hk/citations?user=muMBlr8AAAAJ&hl=zh-TW&oi=sra), X. Yu, [W.C. Xu](https://s2.sci-hub.org.cn/extdomains/scholar.google.com.hk/citations?user=kA3IQHwAAAAJ&hl=zh-TW&oi=sra), T. Salas. H. Smallidge, M. Zhou, H.M. Luo, La_0.8_Sr_0.2_MnO_3_-based perovskite nanoparticles with the A-site deﬁciency as high performance bifunctional oxygen catalyst in alkaline solution., ACS Applied Materials & Interfaces 9 (2017) 23820-23827.

[W. Zhou](https://s2.sci-hub.org.cn/extdomains/scholar.google.com.hk/citations?user=8DuUgRAAAAAJ&hl=zh-TW&oi=sra), [J. Sunarso](https://s2.sci-hub.org.cn/extdomains/scholar.google.com.hk/citations?user=JSh2xRwAAAAJ&hl=zh-TW&oi=sra), Enhancing bi-functional electrocatalytic activity of perovskite by temperature shock: a case study of LaNiO_3−δ_, Journal of Physical Chemistry Letters 4 (2013) 2982-2988.

[X. Xu](https://s2.sci-hub.org.cn/extdomains/scholar.google.com.hk/citations?user=Z92NcLUAAAAJ&hl=zh-TW&oi=sra), [C. Su](https://s2.sci-hub.org.cn/extdomains/scholar.google.com.hk/citations?user=m0hg_PUAAAAJ&hl=zh-TW&oi=sra), [W. Zhou](https://s2.sci-hub.org.cn/extdomains/scholar.google.com.hk/citations?user=8DuUgRAAAAAJ&hl=zh-TW&oi=sra), [Y.L. Zhu](https://s2.sci-hub.org.cn/extdomains/scholar.google.com.hk/citations?user=_KF1bzMAAAAJ&hl=zh-TW&oi=sra), Y.B. Chen, Z.P. Shao, Co‐doping strategy for developing perovskite oxides as highly efficient electrocatalysts for oxygen evolution reaction, Advanced Science 3 (2016) 1500187.

Y. Zhu, W. Zhou, Z.G. Chen, Y.B. Chen, C. Su, M.O.Tadé, Z.P.Shao, SrNb_0.1_Co_0.7_Fe_0.2_O_3−δ_ perovskite as a next‐generation electrocatalyst for oxygen evolution in alkaline solution, Angewandte Chemie (International ed) 54 (2015) 3969-3973.

J.I. Jung, H.Y. Jeong, [M.G. Kim](https://s2.sci-hub.org.cn/extdomains/scholar.google.com.hk/citations?user=XyKqyicAAAAJ&hl=zh-TW&oi=sra), [G. Nam](https://s2.sci-hub.org.cn/extdomains/scholar.google.com.hk/citations?user=cTDLZIoAAAAJ&hl=zh-TW&oi=sra), [J. Park](https://s2.sci-hub.org.cn/extdomains/scholar.google.com.hk/citations?user=E_9UcSoAAAAJ&hl=zh-TW&oi=sra), J. Cho, Fabrication of Ba_0.5_Sr_0.5_Co_0.8_Fe_0.2_O_3–_*_δ_* catalysts with enhanced electrochemical performance by removing an inherent heterogeneous surface film layer, Advanced Materials 27 (2015) 266-271.

[B.Q. Li](https://s2.sci-hub.org.cn/extdomains/scholar.google.com.hk/citations?user=VcOvavIAAAAJ&hl=zh-TW&oi=sra), Z.J. Xia, [B.S. Zhang](https://s2.sci-hub.org.cn/extdomains/scholar.google.com.hk/citations?user=yP4i6R4AAAAJ&hl=zh-TW&oi=sra), [C. Tang](https://s2.sci-hub.org.cn/extdomains/scholar.google.com.hk/citations?user=dvAOfjAAAAAJ&hl=zh-TW&oi=sra), [H.F. Wang](https://s2.sci-hub.org.cn/extdomains/scholar.google.com.hk/citations?user=XgFeackAAAAJ&hl=zh-TW&oi=sra), Q. Zhang, Regulating p-block metals in perovskite nanodots for efﬁcient electrocatalytic water oxidation, Nature Communications 8 (2017) 1177-1185.

[B.Q. Li](https://s2.sci-hub.org.cn/extdomains/scholar.google.com.hk/citations?user=VcOvavIAAAAJ&hl=zh-TW&oi=sra), [C. Tang](https://s2.sci-hub.org.cn/extdomains/scholar.google.com.hk/citations?user=dvAOfjAAAAAJ&hl=zh-TW&oi=sra), [H.F Wang](https://s2.sci-hub.org.cn/extdomains/scholar.google.com.hk/citations?user=XgFeackAAAAJ&hl=zh-TW&oi=sra), X.L Zhu, Q. Zhang, An aqueous preoxidation method for monolithic perovskite electrocatalysts with enhanced water oxidation performance, Science Advances 2 (2016) e1600495.

D.X. Zhen, [B. Zhao](https://s2.sci-hub.org.cn/extdomains/scholar.google.com.hk/citations?user=I7blZhIAAAAJ&hl=zh-TW&oi=sra), H.C. Shin, [Y.F. Bu](https://s2.sci-hub.org.cn/extdomains/scholar.google.com.hk/citations?user=fLZ-lv0AAAAJ&hl=zh-TW&oi=sra), Y. Ding, G.H. He, M.L. Liu, Electrospun porous perovskite La_0.6_Sr_0.4_Co_1–x_Fe_x_O_3–δ_ nanofibers for efficient oxygen evolution reaction, Advanced Materials Interfaces 4 (2017) 1700146.

[Y.L. Zhu](https://s2.sci-hub.org.cn/extdomains/scholar.google.com.hk/citations?user=_KF1bzMAAAAJ&hl=zh-TW&oi=sra), [W. Zhou](https://s2.sci-hub.org.cn/extdomains/scholar.google.com.hk/citations?user=8DuUgRAAAAAJ&hl=zh-TW&oi=sra), [J. Sunarso](https://s2.sci-hub.org.cn/extdomains/scholar.google.com.hk/citations?user=JSh2xRwAAAAJ&hl=zh-TW&oi=sra), [Y.J. Zhong](https://s2.sci-hub.org.cn/extdomains/scholar.google.com.hk/citations?user=UfjTDzIAAAAJ&hl=zh-TW&oi=sra), Z.P. Shao, Phosphorus‐doped perovskite oxide as highly efficient water oxidation electrocatalyst in alkaline solution, Advanced Functional Materials 26 2016 5862-5872.

R.C Liu, F.L Liang, [W. Zhou](https://s2.sci-hub.org.cn/extdomains/scholar.google.com.hk/citations?user=8DuUgRAAAAAJ&hl=zh-TW&oi=sra), Y. Yang, Z.H. Zhu, Calcium-doped lanthanum nickelate layered perovskite and nickel oxide nano-hybrid for highly efficient water oxidation, Nano Energy 12 2015 115-122.

Z.D. Wang, [Y. You](https://s2.sci-hub.org.cn/extdomains/scholar.google.com.hk/citations?user=RJkwhs4AAAAJ&hl=zh-TW&oi=sra), J. Yuan, Y.X. Yin, [Y.T. Li](https://s2.sci-hub.org.cn/extdomains/scholar.google.com.hk/citations?user=fqR_UVYAAAAJ&hl=zh-TW&oi=sra), S. Xin, D.W. Zhang, Nickel-doped La_0.8_Sr_0.2_Mn_1–x_Ni_x_O_3_ nanoparticles containing abundant oxygen vacancies as an optimized bifunctional catalyst for oxygen cathode in rechargeable lithium–air batteries, ACS Applied Materials & Interfaces 8 2016 6520-6528.

[C. Jin](https://s2.sci-hub.org.cn/extdomains/scholar.google.com.hk/citations?user=apMPebsAAAAJ&hl=zh-TW&oi=sra), Z.B. Yang, X.C. Cao, F.L. Lu, R.Z Yang, A novel bifunctional catalyst of Ba_0.9_Co_0.5_Fe_0.4_Nb_0.1_O_3−δ_ perovskite for lithium–air battery, International Journal of Hydrogen Energy 39 2014 2526-2530.

[W. Zhou](https://s2.sci-hub.org.cn/extdomains/scholar.google.com.hk/citations?user=8DuUgRAAAAAJ&hl=zh-TW&oi=sra), M.W. Zhao, F.L Liang, [S.C. Smith](https://s2.sci-hub.org.cn/extdomains/scholar.google.com.hk/citations?user=_oG_CgcAAAAJ&hl=zh-TW&oi=sra), Z.H. Zhu, High activity and durability of novel perovskite electrocatalysts for water oxidation, Materials Horizons 2 (2015) 495-501.

[Y.L. Zhu](https://s2.sci-hub.org.cn/extdomains/scholar.google.com.hk/citations?user=_KF1bzMAAAAJ&hl=zh-TW&oi=sra), [W. Zhou](https://s2.sci-hub.org.cn/extdomains/scholar.google.com.hk/citations?user=8DuUgRAAAAAJ&hl=zh-TW&oi=sra), Y.J. Zhong, Y.F. Bu, X.Y. Chen, Q. Zhong, M.L. Liu, Z.P. Shao, A perovskite nanorod as bifunctional electrocatalyst for overall water splitting. Advanced Energy Materials 7 (2017) 602122.
